# pH-sensitive packaging of cationic particles by an anionic block copolymer shell

**DOI:** 10.1186/s12951-022-01528-0

**Published:** 2022-07-16

**Authors:** Jana I. Solomun, Liam Martin, Prosper Mapfumo, Elisabeth Moek, Elias Amro, Friedrich Becker, Stefan Tuempel, Stephanie Hoeppener, K. Lenhard Rudolph, Anja Traeger

**Affiliations:** 1 Laboratory of Organic and Macromolecular Chemistry (IOMC), Friedrich Schiller University Jena, Humboldtstrasse 10, 07743 Jena, Germany; 2 Jena Center for Soft Matter (JCSM), Friedrich Schiller University Jena, Philosophenweg 7, 07743 Jena, Germany; 3 Leibniz Institute on Aging - Fritz Lipmann Institute (FLI), Beutenbergstraße 11, 07745 Jena, Germany

**Keywords:** Biocompatibility, Charge masking, Core–shell nanoparticles, Layer-by-layer, Stealth polymers

## Abstract

Cationic non-viral vectors show great potential to introduce genetic material into cells, due to their ability to transport large amounts of genetic material and their high synthetic versatility. However, designing materials that are effective without showing toxic effects or undergoing non-specific interactions when applied systemically remains a challenge. The introduction of shielding polymers such as polyethylene glycol (PEG) can enhance biocompatibility and circulation time, however, often impairs transfection efficiency. Herein, a multicomponent polymer system is introduced, based on cationic and hydrophobic particles (P(*n*BMA_46_-*co*-MMA_47_-*co*-DMAEMA_90_), (PBMD)) with high delivery performance and a pH-responsive block copolymer (poly((N-acryloylmorpholine)-*b*-(2-(carboxy)ethyl acrylamide)) (P(NAM_72_-*b*-CEAm_74_), PNC)) as shielding system, with PNAM as alternative to PEG. The pH-sensitive polymer design promotes biocompatibility and excellent stability at extracellular conditions (pH 7.4) and also allows endosomal escape and thus high transfection efficiency under acidic conditions. PNC shielded particles are below 200 nm in diameter and showed stable pDNA complexation. Further, interaction with human erythrocytes at extracellular conditions (pH 7.4) was prevented, while acidic conditions (pH 6) enabled membrane leakage. The particles demonstrate transfection in adherent (HEK293T) as well as difficult-to-transfect suspension cells (K-562), with comparable or superior efficiency compared to commercial linear poly(ethylenimine) (LPEI). Besides, the toxicity of PNC-shielded particles was significantly minimized, in particular in K-562 cells and erythrocytes. In addition, a pilot in vivo experiment on bone marrow blood cells of mice that were injected with PNC-shielded particles, revealed slightly enhanced cell transfection in comparison to naked pDNA. This study demonstrates the applicability of cationic hydrophobic polymers for transfection of adherent and suspension cells in culture as well as in vivo by co-formulation with pH-responsive shielding polymers, without substantially compromising transfection performance.

**Graphical Abstract:**

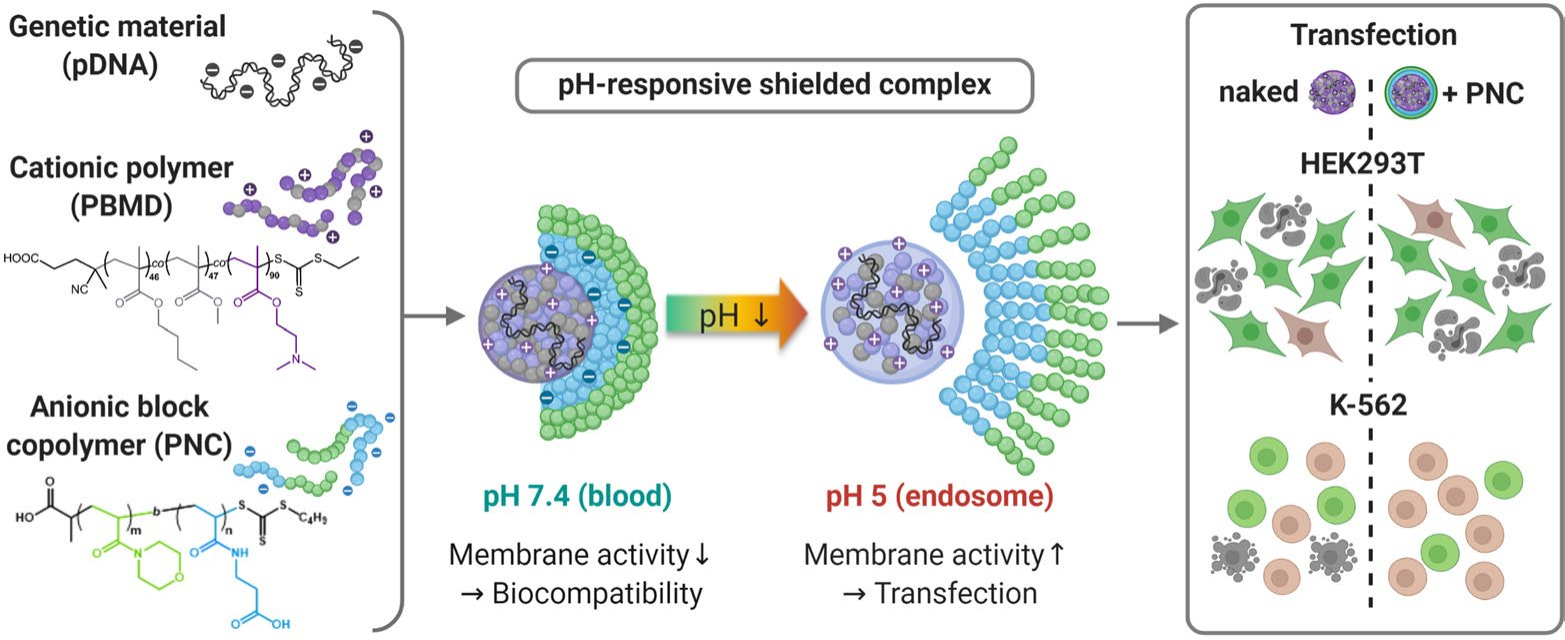

**Supplementary Information:**

The online version contains supplementary material available at 10.1186/s12951-022-01528-0.

## Introduction

Polymers as synthetic gene carriers have been intensively studied for more than a decade. Advantages of cationic polymers include low immunogenic potential in comparison to viral carriers, the ability to bind and condense even high molecular weight genetic material and a high synthetic versatility [1–3]. By electrostatic binding of genetic material, cationic polymers prevent degradation and can facilitate cellular uptake of the hydrophilic and highly negatively charged genetic material via their interaction with cellular membranes [4–6]. However, despite the beneficial effects on transfection efficiency, a high density of cationic moieties is often accompanied with membrane disruptive properties, causing hemolytic and cytotoxic effects [7–9], or is influencing biodistribution of nanomedicines due to opsonization [10–13]. For in vivo applications, the cationic surface charge can be masked by the introduction of hydrophilic shielding polymers. The most prominently used shielding polymer is the well-known FDA approved polyethylene glycol (PEG) [11, 13–15]. However, due to its wide use in medical and cosmetic products, a large number of patients exhibit preexisting antibodies against PEG or generate antibodies as response to a first dose of PEGylated nanomaterials, potentially leading to premature and rapid clearance of PEGylated nanocarriers and their accumulation in liver and spleen. This so-called accelerated blood clearance (ABC) effect results in shorter circulation times and potentially increases cytotoxicity, whereas the presence of anti-PEG antibodies can further cause allergic reactions and severe side effects in patients [16–19]. The recently developed mRNA-based vaccines against SARS-CoV-2 involving PEGylated lipids are some of the latest examples where PEG is suspected of causing allergic reactions in some patients, necessitating the development of alternative shielding polymers [20, 21]. A promising alternative to PEG is Poly(*N*‐acryloylmorpholine) (PNAM), a hydrophilic polymer that exhibits low cytotoxicity and reduced protein interaction, leading to reduced immunogenic potential and improved hemocompatibility but, unlike PEG, does not induce the ABC effect [22–27].

However, the introduction of shielding polymers tends to impair transfection efficiency of cationic gene delivery vectors due to insufficient condensation of genetic material caused by steric hindrance, reduced cellular uptake and endosomal escape, or insufficient release of genetic material from the polyplex at its site of action [15, 28, 29]. An effective yet biocompatible gene delivery vector should therefore be flexible in its physical properties in order to handle, both, extracellular and intracellular environments.

pH-responsive stealth systems are of particular interest as the pH value in intracellular compartments such as endosomes (pH 5–6) is lower compared to the extracellular environment (pH 7.4) which can be exploited for nanocarrier design. This can be achieved by, *e.g.,* the introduction of acid cleavable linkers between cationic and stealth polymer [30, 31], or non-covalent electrostatic coating by anionic copolymers without the need for synthetic modification of the cationic polymer [32–37]. Non-covalent coating approaches by PEG containing (bio)polymers have been mainly applied to hydrophilic polyplexes such as PEI, with impact on the electrostatic complexation of the genetic material within the polyplex, which can impair transfection efficiency and raise concerns about stability in vivo.[32, 38]

The introduction of hydrophobicity is a known and straight forward approach to improve the colloidal stability of cationic polyplexes in the presence of competing polyanions [39–41]. It can be envisioned that it can also improve the stability of non-covalent shielding approaches. Furthermore, hydrophobic modifications can enhance membrane interactions, facilitating endosomal escape, thus, resulting in increased transfection efficiencies in many cell lines, including difficult-to-transfect primary and suspension cells, *e.g.,* the chronic myeloid leukemia (CML) cell line K-562 [42]. These cell lines are of interest for gene delivery applications such as vaccination, cancer treatment or inflammatory diseases [43, 44]. In our recent work we established a novel aqueous formulation of cationic hydrophobic particles loaded with various classes of genetic material using the hydrophobic cationic polymer P(*n*BMA_46_-*co*-MMA_47_-*co*-DMAEMA_90_) (PBMD). In contrast to commonly studied water-soluble polymers forming polyplexes and micelles, the PBMD polymer is insoluble under physiological conditions (pH 7.4) and results in nanoparticles which only dissolve under acidic conditions. pDNA loaded PBMD-particles are highly stable at physiological pH values (pH 7.4, blood) and in the presence of competing polyanions such as heparin, enabling high transfection efficiencies in HEK293T cells even at low pDNA concentrations [45].

Based on these considerations, we exploit the potential of these stable and highly efficient pDNA encapsulating PBMD particles for a non-covalent, electrostatic and pH-responsive shielding approach, for difficult-to-transfect suspension cells and to improve the in vivo applicability. Therefore, the cationic charge of the pDNA loaded PBMD particles (PBMD(pDNA)) was masked by a diblock copolymer poly((N-acryloylmorpholine)-*b*-(2-(carboxy)ethyl acrylamide)) (P(NAM_72_-*b*-CEAm_74_), PNC). The polymer is composed of an anionic pH-responsive PCEAm block interacting with the tertiary amines of the PBMD polymer as cationic counterpart and a PNAM “stealthy” block as alternative to PEG. The pH-responsiveness of the system was confirmed by titration experiments and demonstrated on a microparticle scale by visual inspection. Following this, pDNA loaded PBMD(pDNA) nanoparticles shielded by the PNC polymer were characterized in terms of pDNA binding, stability in the presence of shielding polymer, size and surface charge, followed by evaluation of metabolic and membrane activity and hemocompatibility with human erythrocytes. Cellular uptake and transfection efficiency in adherent (HEK293T) and difficult-to-transfect suspension cells (K-562) were evaluated, followed by initial in vivo studies investigating the applicability of the system and the delivery to bone marrow blood cells. In summary, these experiments demonstrate the high potential of the pH-triggered shielding system based on cationic hydrophobic particles and its utilization for transfection of cells including hard-to-transfect suspension cells in culture and in vivo.

## Main methods

Materials and further methods such as synthesis of the control polymers, titration, gel retardation assay (GRA), dynamic and electrophoretic light scattering (DLS, ELS), cryo-TEM measurements, cytotoxicity assays cellular uptake and transfection experiments are described in the SI (Additional file 1) [42, 45, 46]. Furthermore, in vivo transfection experiments are described in the SI.

### Synthesis and characterization of P(NAM_72_***-b***-CEAm_74_) via aqueous RAFT polymerization

PABTC (45.0 mg, 1.89 × 10^–4^ mol), NAM (2000.1 mg, 1.42 × 10^–2^ mol), ultrapure water (1094.8 mg), 1,4-dioxane (270.1 mg), a 0.1% (w/w) solution of 2,2′-Azobis(2-(2-imidazolin-2-yl)propane)dihydrochloride (VA-044) in ultrapure water (398.9 mg, 0.399 mg VA-044, 1.23 × 10^–6^ mol) and 1,3,5-trioxane (external NMR standard, 26.6 mg) were introduced to a 4 mL microwave vial equipped with a magnetic stirring bar. The vial was sealed, and the solution deoxygenated by bubbling argon through it for approx. 10 min. The vial was placed in an oil bath set at 70 °C and allowed to stir for 2 h. The vial was then cooled and opened, and samples were taken for NMR and SEC analysis. Monomer conversion: ≥ 96%, DMAc-SEC: *M*_n,SEC_ = 10.4 kg mol^−1^, *Ð* = 1.09. A portion of the crude PNAM_72_ (906.0 mg, 4.46 × 10^–5^ mol polymer) was transferred to a 4 mL microwave vial equipped with a magnetic stirring bar. CEAm (478.8 mg, 3.35 × 10^–3^ mol), ultrapure water (982.8 mg), a 0.1% (w/w) solution of VA-044 in ultrapure water (471.9 mg, 0.47 mg VA-044, 1.46 × 10^–6^ mol) and additional 1,3,5-trioxane (18.3 mg) was added, the vial was sealed, the solution deoxygenated with argon, and placed in an oil bath set at 70 °C. Samples were taken for NMR and SEC analysis. The polymer was dialyzed against deionized water for 4 days (MWCO: 3.5 kDa) and lyophilized to give a pale-yellow solid. Aq-SEC: *M*_n,SEC_ = 27,360 kg mol^−1^, *Ð* = 1.21.

### Preparation and investigation of layered microparticles

For microparticle preparation, the PBMD polymer was dissolved in 250 µL dichloromethane (DCM) (20 mg mL^−1^). Neutral lipid orange (NLO) was prepared as a stock solution in DCM (1 mg mL^−1^) and 2.5 µL was added to the PBMD solution. Subsequently the PBMD-NLO solution was added to 5 mL of a 0.3% (w/v) solution of PVA and rapidly mixed with an ULTRA-TURRAX T25 homogenizer (IKA, Staufen im Breisgau, Germany) at a speed of 8000 rpm for 10 s. The organic solvent was allowed to evaporate while stirring at 800 rpm for 24 h. For CLSM studies PNC polymer labeled with DY-635 (PNC_DY635_) was dissolved in ddH_2_O (10 mg mL^−1^) and added to the microparticles at a molar ratio of shielding polymer (CEAm or NAM) to the cationic DMAEMA groups of the PBMD polymer (COOH/NH ratio, in the following referred to as layer to core ratio, L/C ratio) of 0.6. For calculations of the L/C ratios the calculated *M*_n,th_ was used. For investigations of the pH-dependent behavior of the PBMD microparticles (PBMD_MP_) shielded with PNC_DY635_ the samples were diluted with buffer at the respective pH value at a ratio of 1:4 (HBG buffer pH 7.4 (5% (w/v) glucose, 20 mM HEPES; acetate buffer pH 5 and 6) or fetal bovine serum (FBS) to investigate the behavior in the presence of serum proteins. The samples were imaged using a LSM880, Elyra PS.1 system (Zeiss, Jena, Germany). NLO was excited at 488 nm by applying the argon laser (3%) using emission filters for 507–581 nm with a gain of 800. For excitation of DY-635 the Helium Neon (He–Ne) laser (3%) at 633 nm was used with a detection filter for 654–759 nm and a gain of 680. To avoid cross talk NLO and DY-635 were imaged in two separate tracks using the frame scan modus. For magnification, a 63 × 1.4 DIC plan apochromat oil objective was used. Images were acquired using the ZEN software, version 2.3 SP1 (Zeiss, Jena, Germany) and were processed using the ZEN software and ImageJ (Version 1.52a, National Institutes of Health, Bethesda, MD, U.S.).

### Formulation of layered nanoparticles

Cationic pDNA-loaded particles were prepared by a slightly adjusted pH-dependent formulation method previously described [45]. Briefly, a stock solution of the PBMD polymer in 0.2 M sodium acetate buffer (pH 5.8) was diluted with 5% (w/v) glucose solution to obtain concentrations that result in a nitrogen to phosphate ratio (N/P ratio) of 10 within the particle and mixed with pDNA at a ratio of 1:2. The samples were vortexed for 10 s and incubated for 5 min at room temperature (RT) prior to the addition of the shielding (PNC) or control (PNAM, PCEAm) polymers. Stock solutions of the polymers were prepared by dissolution in 5% (w/v) glucose (10 mg mL^−1^). For preparation of shielded particles, the shielding and control polymers were diluted in 5% (w/v) glucose solution to obtain varying L/C ratios. Subsequently the pDNA loaded PBMD particles (PBMD(pDNA)) were added to the shielding or control polymer solution by slowly pipetting up and down. The shielded particles were incubated for 15 min at RT before usage.

### Ethidium bromide binding assay (EBA) and heparin release assay (HRA)

The stability of pDNA complexation after addition of PNC or the control polymers was further investigated in detail by using an ethidium bromide (EtBr) quenching assay [46]. For sample preparation, pKMyc-pDNA at a concentration of 15 µg mL^−1^ was incubated with EtBr in 5% (w/v) glucose for 10 min. Subsequently, shielded pDNA loaded PBMD(pDNA) particles were prepared as described above. In a black 96-well plate (Nunc, Thermo Fisher, Waltham, MA, U.S.) the samples were diluted 1:2 with buffer solutions to reach the desired pH values (HBG pH 7.4, acetate pH 5, pH 6) and incubated for 15 min at 37 °C. EtBr fluorescence intensity was measured at *λ*Ex = 525 nm/*λ*Em = 605 nm. pDNA without polymer diluted in the respective buffer solution was defined as 100% free DNA and the relative fluorescence intensity of the samples (RFI) was calculated according to Eq. 1.

1 $$\mathrm{RFI }/=\mathrm{ \%}\frac{{\mathrm{FI}}_{\mathrm{Sample}}}{{\mathrm{FI}}_{\mathrm{pDNA}}} \cdot 100$$

where FI_sample_ and FI_pDNA_ represent the fluorescence intensity of the sample and pDNA without polymer, respectively.

The release of complexed pDNA was studied as described before. Briefly, heparin was added stepwise to naked and shielded particles (L/C 0.6) and the resulting changes in EtBr fluorescence intensity were measured. Influence of the pH value on pDNA binding and release was studied by performing the assay at varying pH values as described for the EBA. The obtained measurement points of EBA and HRA were fitted using OriginPro Software (Version 2018b, Origin Lab Corporation, Northampton, MA, U.S.) using a b-spline function to represent the apparent experimental results as a guide to the eye.

### Cell culture

The human embryonic kidney cell line HEK293T was cultured in Dulbecco’s modified eagle medium (DMEM, 1 g L^−1^ glucose, supplemented with 10% (v/v) FBS, 100 U mL^−1^ penicillin, 100 µg mL^−1^ streptomycin) (D10). The chronic myeloid leukemia cell line K-562 was cultured in Roswell Park Memorial Institute (RPMI) 1640 medium supplemented with 10% (v/v) FBS, 100 U mL^−1^ penicillin and 100 µg mL^−1^ streptomycin (R10). Both cell lines were cultured at 37 °C in a humidified 5% (v/v) CO_2_ atmosphere. For experiments HEK293T cells were seeded into 24-well plates at a density of 0.2 × 10^6^ cells mL^−1^ in 500 µL D10 supplemented with 10 mM HEPES (D10 + H) and incubated for 24 h at 37 °C in a humidified 5% (v/v) CO_2_ atmosphere. Unless stated otherwise the medium of the HEK293T cells was changed to 450 µL fresh D10 + H 1 h prior to the start of experiments. K-562 cells were seeded into cell culture flasks in a density of 0.3 × 10^6^ cells mL^−1^ in 8 mL R10 one day prior to experiments. On the day of experiments the cells were seeded into 24-well plates at a density of 0.3 × 10^6^ cells mL^−1^ in 500 µL R10 supplemented with 10 mM HEPES (R10 + H) 3 h prior to treatment.

### Hemolysis assay with human erythrocytes

The interaction of the polymers with human erythrocytes was investigated as published before [46]. Citrate blood from human donors was obtained from the Department of Transfusion Medicine of the University Hospital Jena. For separation of the blood cells the blood was centrifuged at 4500 ×*g* for 5 min, the supernatant was removed, and the cells were resuspended in phosphate buffered saline (PBS, pH 7.4). These steps were repeated twice more. After the final centrifugation step the cells were resuspended in PBS (pH 6 or pH 7.4). The shielded PBMD(pDNA) particles were diluted with PBS (pH 6 or pH 7.4) to the respective concentration in a volume of 350 µL and mixed with the resuspended cells at a ratio of 1:1. After incubation at 37 °C for 1 h the samples were centrifuged at 2400 ×*g* for 5 min and the supernatant was transferred to a 96-well plate. Hemoglobin release was determined by measuring the absorbance at 555 nm in a plate reader (Infinite M200 PRO microplate reader, Tecan, Männedorf, Switzerland). Absorbance at 630 nm was measured as reference. Cells incubated with a 1% Triton-X-100 solution were used as positive control and stated as 100% hemolysis. PBS was used as negative control and stated as 0% hemolysis. Experiments were conducted with blood from three different donors in technical triplicates. The hemolytic activity was calculated according to Eq. 2

2 $$\mathrm{Hemolysis}/\mathrm{\%}= \frac{\left({\mathrm{A}}_{\mathrm{sample}}- {\mathrm{A}}_{\mathrm{neg}.\mathrm{ control}}\right)}{{(\mathrm{A}}_{\mathrm{pos}.\mathrm{ control}}-{\mathrm{A}}_{\mathrm{neg}.\mathrm{ control}})}\cdot 100$$

where A_Sample_, A_neg. control_ and A_pos. control_ are the absorption values of a given sample, the PBS treatment and the Triton X-100 treatment, respectively.

### Statistical analysis

To determine statistically significant differences, multiple groups were either analyzed by two-way mixed analysis of variance (two-way mixed ANOVA) or one-way ANOVA followed by Bonferroni’s post-hoc test. Experiments on the comparison of two groups in total were analyzed by the unpaired t-Test. Statistical significance is denoted as follows: */#p < 0.05, **/##p < 0.01, and ***/###p < 0.001 and analysis was conducted using OriginPro2018b software.

## Results and discussion

### Synthesis, characterization and pH-responsiveness of layer polymers

The block copolymer P(NAM-*b*-CEAm) (PNC) was synthesized via RAFT polymerization targeting an overall degree of polymerization (DP_*n*_) of 75 for the PNAM block. The polymerization kinetics were followed using ^1^H-NMR spectroscopy and size exclusion chromatography (SEC) in DMAc (DMAc-SEC) (Table 1) and revealed a monomer conversion of ≥ 96% and a narrow mass distribution of *Ð* = 1.09 with a DP_*n*_ of 72. PNAM_72_ was subsequently chain extended with CEAm. The purified polymer possessed an overall DP_*n*_ of 146 based on ^1^H-NMR, with a molar mass (*M*_n,SEC_) of 27.4 kg mol^−1^ and a narrow molar mass distribution of *Ð* = 1.21 (Additional file 1: Fig. S1 and S2, Table S1). Homopolymers composed of a similar number of NAM or CEAm monomers per chain as the PNC polymer, served as control polymers (Table 1, Additional file 1: Fig. S3).

**Table 1:** Overview of polymers used within the study. Polymers were characterized by SEC and the *pK*_*a*_ was determined by titration experiments

Label	Polymer	DP_NAM_	DP_CEAm_	*M*_n,th_	*M*_n,SEC_	*Ð*	*pK*_*a*_
PNC	pNAM-*b*-pCEAm	72	74	21,350	27,360^b^	1.21	5.2
PNAM	pNAM	72	–	10,200	8600^a^	1.12	–
PCEAm	pCEAm	–	72	10,500	18,000^b^	1.36	5.1

^a^DMAc-SEC (DMAc + 0.21% LiCl) with PMMA standards

^b^Aq-SEC (0.1 M NaNO_3_/0.05% NaN_3_) with PEG standards

**Fig. 1:**
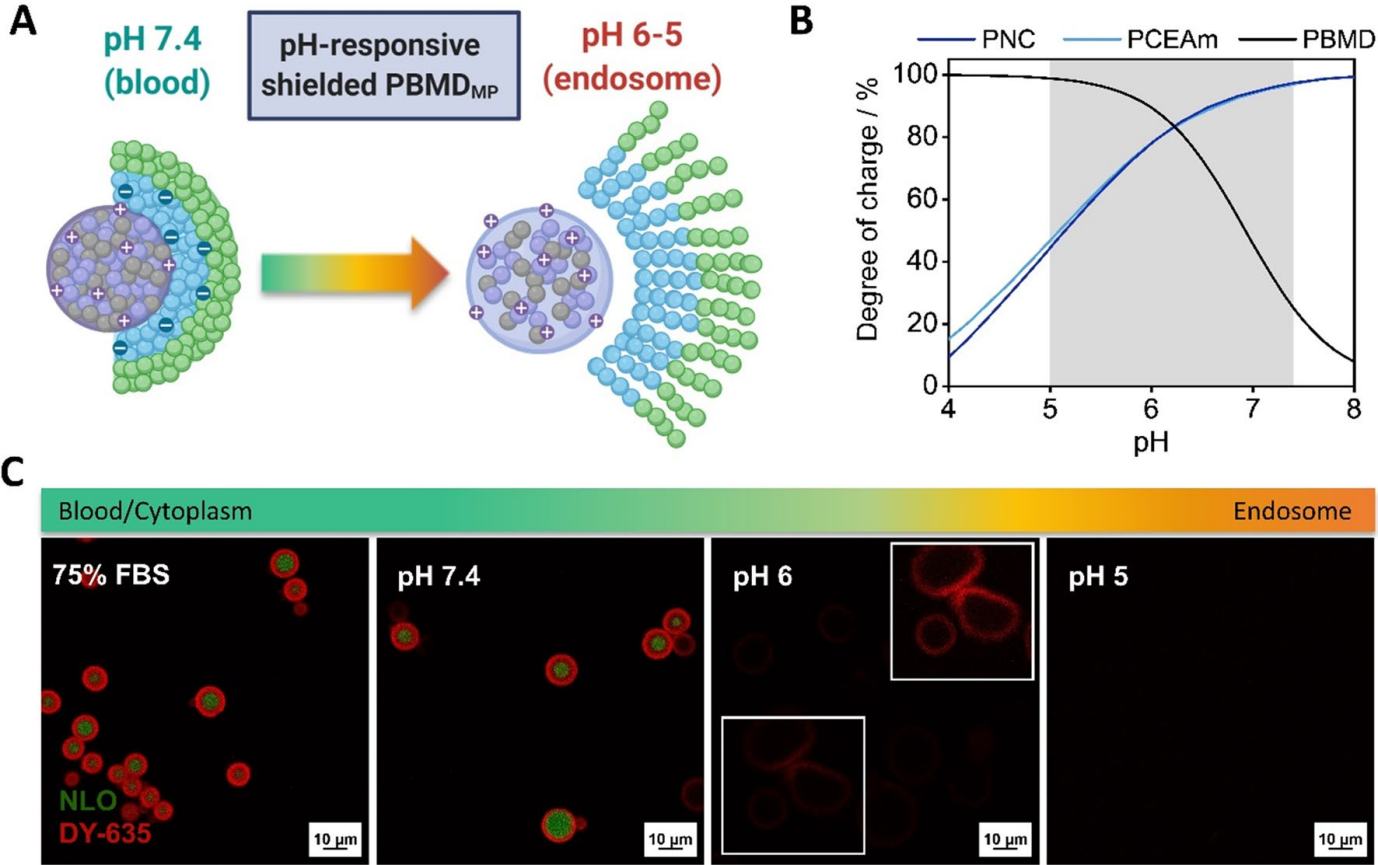
Principle of shielded microparticle system and visualization by confocal laser scanning microscopy (CLSM). **A** Scheme of pH-responsive behavior of the shielded microparticle system. This figure was created with Biorender.com. **B** Degree of charge calculation of PCEAm and P(pNAM-*b*-pCEAm) (PNC) within the physiological relevant pH range (5–7.4) highlighted in grey. **C** CLSM investigation of PBMD-microparticles (PBMD_MP_) encapsulating neutral lipid orange (NLO, green) prepared with PNC_DY635_ (red) incubated in different buffers (HBG pH 7.4, acetate pH 6, 5) and FBS for > 15 min. The upper inset in (**C**) display a section of the same picture without in which the contrast was enhanced

To evaluate the pH-responsiveness of the PNC polymer and the PCEAm homopolymer, titration experiments were conducted (Additional file 1: Fig. S5). When compared to the *pK*_*a*_ of poly(acrylic acid) (PAA) the calculated *pK*_*a*_ values of the PNC and PCEAm polymer (*pK*_*a*, PNC_ = 5.2; *pK*_*a*,PCEAm_ = 5.1) were slightly higher, potentially due to the ethyl linker between acrylate backbone and carboxylic acid group [47–49]. Calculations of the degree of charge based on the titration data (ratio between protonated units to total units estimated for each pH value) clearly demonstrated the pH-responsive charge behavior of the polymers. The PNC polymer is highly charged at neutral pH values (97% at pH 7.4, Fig. 1B), while with decreasing pH values charge is reduced (46% at pH 5.1). As known from our previous work, the PBMD polymer precipitates from the titration solution at higher pH values. Therefore, a hypothetical degree of charge was directly calculated from the apparent *pK*_*a*_ (6.9) determined previously [45]. The PBMD polymer shows a charge behavior inverse to the PNC polymer; being highly charged at acidic pH, while charge is reduced at neutral pH values. Therefore, under neutral conditions, the strongly charged anionic PNC polymer can interact electrostatically with the cationic PBMD polymer. A decrease to endosomal pH values will result in reduced interaction, due to decreased charge of the PNC polymer (Fig. 1A). To microscopically visualize the pH-responsiveness and stability of the electrostatic interaction between the polymers in the presence of serum, PBMD microparticles (PBMD_MP_) encapsulating the hydrophobic and solvatochromic dye neutral lipid orange (NLO) were prepared and shielded with a DY-635 labelled PNC polymer (PNC_DY-635,_ characterization shown in Additional file 1: Fig. S4). The shielded microparticles were incubated with either fetal bovine serum (FBS) or buffers in the pH range from 7.4 to 5 (blood to endosomal pH values). Microscopic investigations of the microparticles revealed a clearly visible layered structure with the PNC_DY635_ polymer surrounding the PBMD_MP_ core at pH 7.4 (Fig. 1C, Additional file 1: Fig. S6). Further, the particles remained stable after incubation with 75% (v/v) FBS, revealing no signs of aggregation or dissolution. Acidification to pH 6 resulted in an increase in size and a loss of NLO fluorescence in the core of the PBMD_MP_, which can be attributed to a more hydrophilic environment due to increased cationic charges associated with dissolution and swelling of the particle. At pH 5 complete dissolution of the microparticles was observed. Based on these observations it can be concluded that the shielded microparticles demonstrated a stable interaction between the PBMD core and the PNC shielding polymer under neutral pH conditions (7.4, blood) and in the presence of serum, showing no signs of premature dissolution or aggregation, while dissolution and swelling behavior, indicating reduced interaction, occurred during acidification. Overall, this pH-responsive interaction behavior renders the polymer system a promising candidate for application to pDNA loaded nanoparticles and their systemic administration.

### Particle stability and pDNA binding

**Fig. 2:**
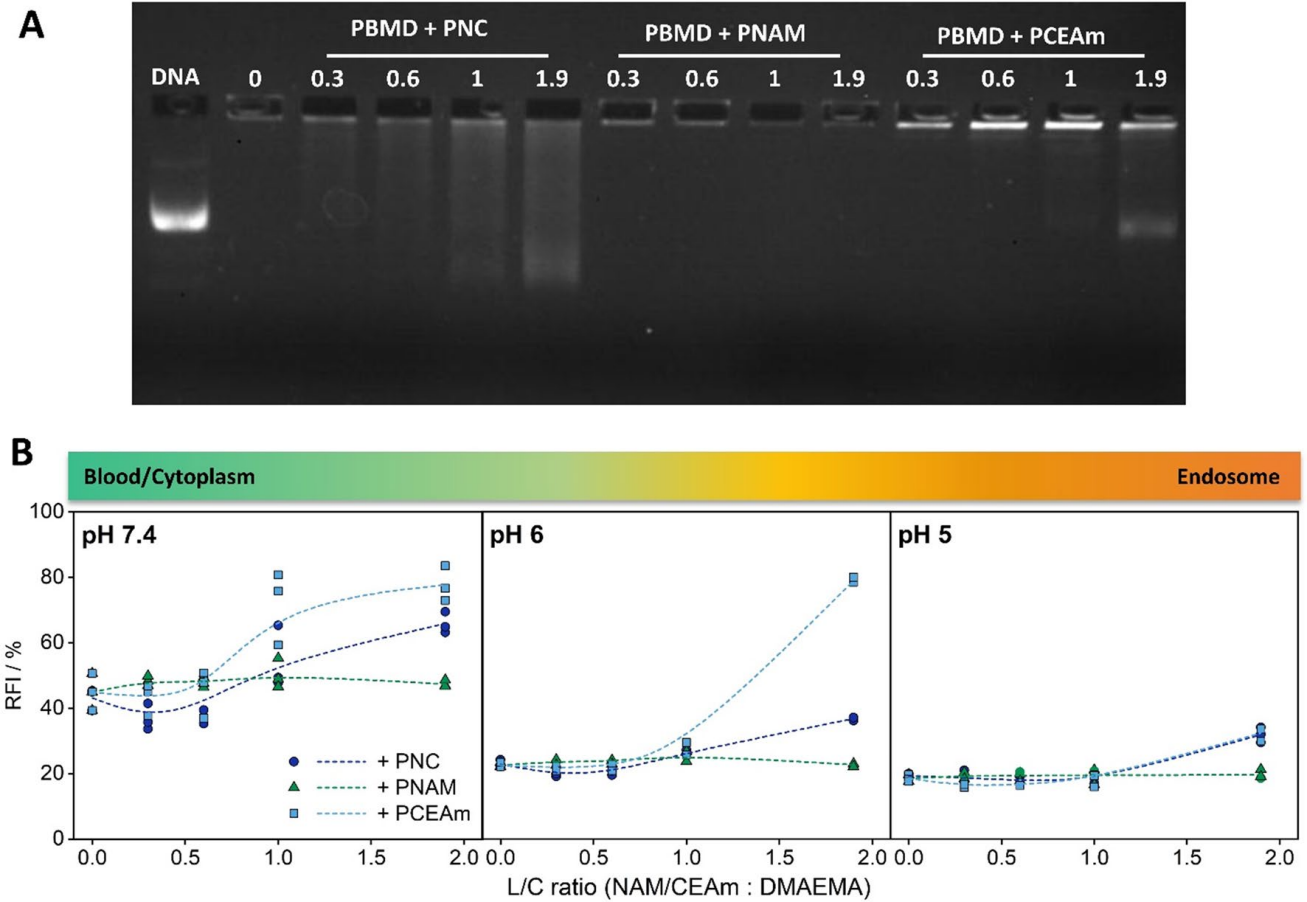
Influence of the amount of layer polymer on the stability of pDNA complexation by the PBMD polymer. pDNA binding after addition of increasing amount of layer polymer was evaluated by **A** gel electrophoresis (DNA: pDNA, 0: naked PBMD(pDNA) particle) and **B** ethidium bromide binding assay (EBA) at different pH values (HBG buffer pH 7.4, acetate buffer pH 6 and 5) at N/P 10 (n = 3). The obtained data were fitted using a b-spline function

Following the evaluation of the pH-responsive behavior of the polymer system itself, the applicability of the shielding principle to pDNA loaded PBMD (PBMD(pDNA)) particles was studied. Although the PBMD(pDNA) particle is stabilized by hydrophobic interactions, the complexation of pDNA is still partially driven by electrostatic interactions, and therefore potentially subject to interference by competing polyanions such as the PNC polymer. In order to evaluate the stability of pDNA binding a gel retardation assay (GRA) was performed for qualitative assessment [50], while the ethidium bromide binding assay (EBA) allowed a quantitative examination [51]. For both assays, increasing amounts of the PNC polymer were added to the PBMD(pDNA) particle, based on the calculated molar ratio of CEAm or NAM to cationic DMAEMA groups of the PBMD polymer (COOH/NH ratio, in the following referred to as layer to core ratio, L/C ratio). The PCEAm and PNAM homopolymer, were used as controls. Within the GRA full complexation of pDNA in PBMD(pDNA) particles at N/P 10 was observed (Fig. 2A, L/C ratio 0). In both assays, addition of PNAM homopolymer to the PBMD(pDNA) particles had no influence on pDNA complexation, which is in line with the assumption of no or weak interaction of the uncharged hydrophilic PNAM homopolymer with the PBMD(pDNA) particles. In contrast, PNC and PCEAm varied substantially in their impact on pDNA complexation. The addition of the anionic homopolymer PCEAm had a strong influence on pDNA binding, indicated in the GRA by an increase of ethidium bromide (EtBr) fluorescence intensity in the loading pockets and a clear displacement of pDNA at a L/C ratio of 1.9. In comparison, the addition of the PNC block copolymer resulted in only slight smears, with no clear displacement of pDNA from the particle. This is supported by the EBA, which showed a stronger increase in the relative EtBr fluorescence intensity (RFI) upon addition of the PCEAm homopolymer in comparison to the PNC polymer (Fig. 2B). Interestingly, by addition of the PNC polymer at L/C ratios of ≤ 0.6 a decrease in RFI was observed, potentially indicating a further stabilization of the particle by the PNC polymer. In order to investigate the influence of acidification on pDNA complexation the EBA was additionally performed at pH 6 and 5. RFI values prior to addition of shielding polymers decreased to 20%, showing a clear influence of acidification on the pDNA binding behavior of the PBMD polymer. This is in line with our previous study and may be attributed to changes in the balance between cationic and hydrophobic moieties towards cationic charges within the PBMD polymer [45]. At pH 6 the addition of PNC polymer resulted in reduced displacement of pDNA while the addition of lager amounts (L/C ratio 1.9) of PCEAm homopolymer displaced pDNA from the PBMD(pDNA) particle. A further decrease to pH 5 in general reduced interaction between the PBMD core and the shielding polymers, even at high L/C ratios. Differences in the extent of pDNA displacement by the PNC and PCEAm homopolymer at acidic pH could be attributed to steric constraints. The hydrophilic PNAM block of the PNC polymer is potentially reducing the interaction with the cationic core and therefore prevents replacement of pDNA by the anionic block.

Overall, the anionic polymers PNC and PCEAm are clearly interacting with the PBMD(pDNA) particle at pH 7.4, while PNAM shows no interaction. Furthermore, the introduction of PNAM into the anionic PNC block copolymer seems to prevent displacement of pDNA by the anionic CEAm groups and stabilizes the particle at neutral pH values in comparison to the anionic PCEAm homopolymer. Measurements at lower pH values demonstrated the pH-responsive behavior of the pDNA loaded system and a reduction in interaction between cationic core and PNC shielding polymer, especially at pH 5, underlining the pH-responsive behavior observed microscopically for the NLO loaded microparticles (Fig. 1C). In order to further study particle stability at varying pH values in the presence of competing anions a heparin release assay (HRA) was conducted. Therefore, changes in EtBr fluorescence intensity were studied upon addition of increasing amounts of heparin (Additional file 1: Fig. S7) to naked and shielded PBMD particles (L/C 0.6). Overall there was a clear influence of pH observable. While all particles release pDNA at heparin concentrations higher 9 U mL^−1^ at acidic pH values (6 and 5), only a slight increase in EtBr fluorescence intensity is observed even at the higher heparin concentration (103 U mL^−1^) for all particles at neutral pH values. This pH-dependent release behavior of the PBMD particle showing high stability at neutral pH and release at endosomal pH is in line with our previous studies and is clearly not impaired by the shielding polymers [45]. Thus naked and shielded particles both show high stability at neutral pH and pDNA release at acidic pH values in the presence of anionic components.

### Size and surface charge of shielded PBMD(pDNA) particles

**Fig. 3:**
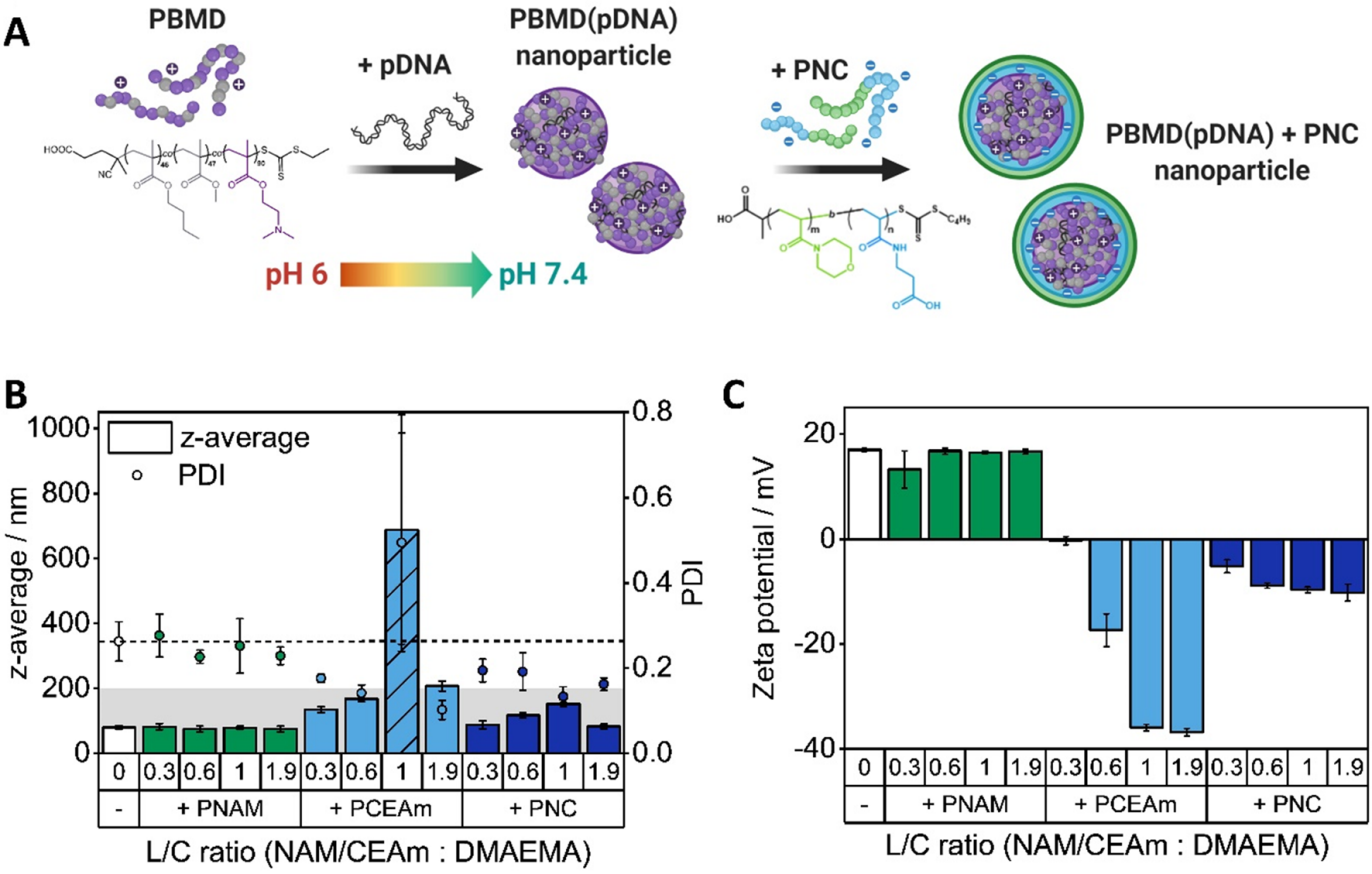
Size and surface charge measurements of shielded PBMD (pDNA) particles. **A** The scheme presents the formulation process of PNC shielded PBMD (pDNA) nanoparticles. **B** Shielded particles (PBMD + PNC) at varying molar ratios were characterized in terms of diameter (z-average) and polydispersity (PDI) by DLS in comparison to the control polymers (PBMD + PNAM, PBMD + PCEAm). Striped bars mark the occurrence of large aggregates (≥ 1000 nm) that were taken into account as 1000 nm to determine the mean value. The gray area indicates the desired size range (≤ 200 nm) and the dashed line the indicates the PDI value obtained for naked PBMD(pDNA) particles (0.26) (mean of n = 3 ± SD). **C** The particles were further characterized in terms of surface charge (zeta potential, mean of n = 3 ± SD)

In addition to the ability to form stable particles with pDNA, particle size and surface charge have been shown to highly impair cellular uptake and therefore gene delivery efficiency. While particle sizes below 200 nm are favored for controlled endocytotic cellular uptake and prolonged blood circulation [28, 52, 53], the surface charge has been shown to have an impact on the extent of cellular uptake by different cell types and systemic distribution [28, 54]. Therefore, layered PBMD(pDNA) particles at varying L/C ratios were characterized by DLS and ELS measurements (Fig. 3, Additional file 1: Fig. S8). In summary, PBMD(pDNA) particles possessed a diameter (z-average) within the favorable range below 200 nm (79.5 ± 5.9 nm) with PDI of 0.262 and a positive surface charge (17 ± 0.3 mV) that was unchanged following the addition of PNAM (Fig. 3), complementing the results from the GRA and EBA and indicating no interaction (Fig. 2). The addition of the PNC polymer slightly increased particle diameter (87.5 to 152.3 nm) but reduced PDI values (0.133 to 0.195), while upon addition of the PCEAm homopolymer particle size increased, with large aggregates forming at an L/C ratio of 1. The surface charge shifted to moderate negative values in the presence of the PNC polymer (– 10.2 mV at an L/C of 1.9), while the PCEAm polymer lead to highly negative surface charge, especially at L/C ratios ≥ 0.6 (-36.8 ± 0.7 mV). This difference could be explained by the NAM block of the PNC polymer potentially reducing the surface charge in comparison to the anionic PCEAm homopolymer. Taking additionally the results of the GRA and EBA (Fig. 2) into account, the strong decrease of the surface charge by the PCEAm homopolymer could also occur due to partial displacement of negatively charged pDNA being subsequently present in the solution. However, the shielding of PBMD(pDNA) particles by the PNC polymer resulted in stable particles in the desirable size range with low PDI values and moderate negative surface charge. For the following experiments, a L/C ratio of 0.6 was selected as the most promising ratio for further applications, as this ratio indicated stable complexation (Fig. 2B) and nicely distributed particles with a moderate negative zeta potential (Fig. 3C).

pH-Responsive size and morphology change of the particles was studied by DLS and cryo-TEM measurements at neutral (7.4) and endosomal pH (5) (Additional file 1: Fig. S9). DLS measurements showed no substantial changes in size and PDI upon pH change. A slight decrease in the correlation function of naked PBMD particles at pH 5 in comparison to pH 7.4 indicated a decrease in particle concentration, whereas no change was observed for PNC shielded particles. Cryo-TEM images of naked PBMD particles after preparation revealed nanostructures below 100 nm which is in line with our previous study [45]. Slightly larger structures were observed for PNC shielded particles which are observed to have also slightly more defined particle boundaries. Adjustment of the pH to 7.4 resulted in slight agglomeration of the particles which was more pronounced for PNC shielded particles and is also indicated in DLS measurements, which could be caused by a reduction of the repulsive forces. At endosomal pH values PBMD particles were not visible anymore by cryo-TEM, whereas PNC shielded particles decreased in number and showed even less defined particle boundaries. This could be either attributed to dissolution of the particles or indicate changes in the particle structure and hydrophobicity. The second hypothesis is supported by DLS measurements obtained at pH 5. Thus, DLS and cryo-TEM measurements at pH 7.4 and 5 reveal the pH-responsive behavior of naked and PNC shielded particles, showing defined nanostructures at pH 7.4 and a morphology change and dissolution at pH 5.

### Evaluation of cytotoxicity and hemocompatibility

**Fig. 4:**
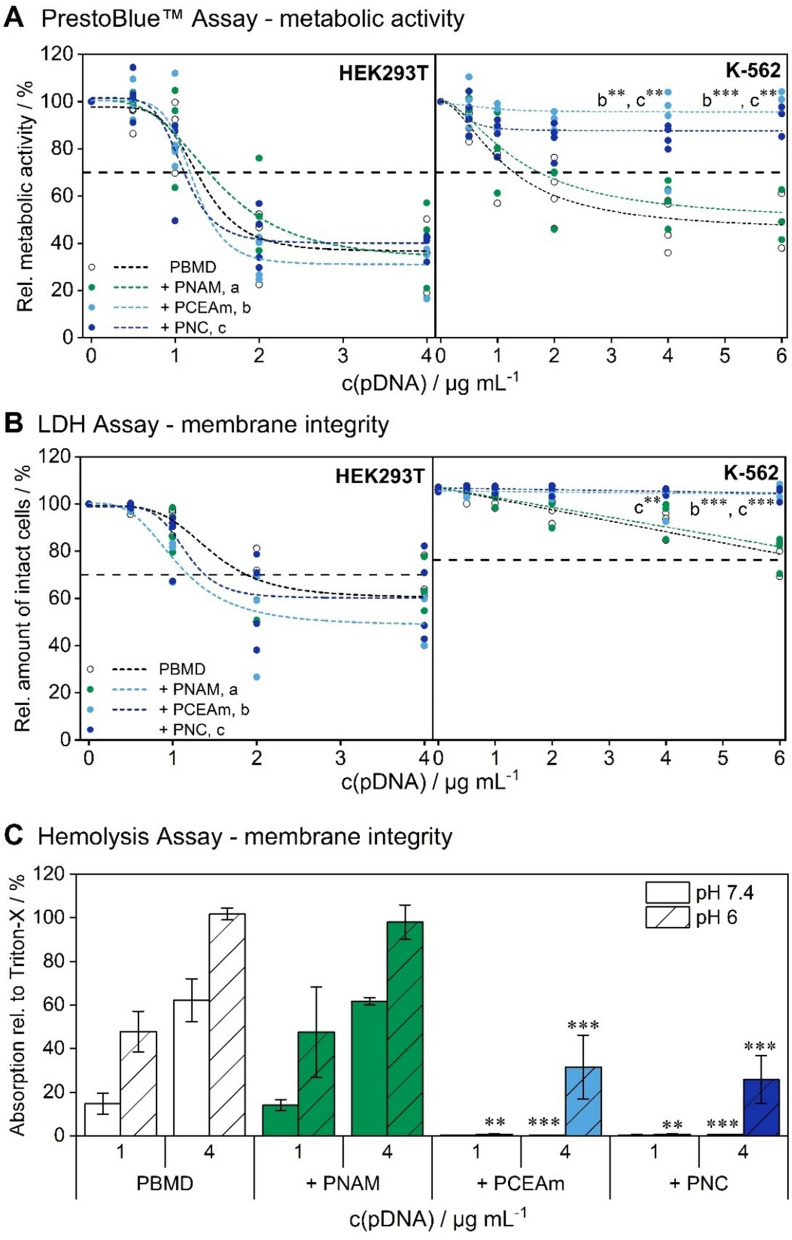
Evaluation of cytotoxicity and hemocompatibility of PBMD(pDNA) particles without (PBMD) and with shielding and control polymers (+ PNC, + PNAM, + PCEAm) at L/C 0.6. Cytotoxicity was investigated by measuring **A** the metabolic activity (PrestoBlue™ assay) and **B** LDH release to evaluate membrane integrity (Cytotox-ONE™ assay). HEK293T cells were incubated for 4 h with the particles and subsequently for 20 h in fresh media, K-562 for 24 h with the particles without media change. Data are presented as single measurement points and were fitted using either logistic or linear functions (n ≥ 3). Statistical significant differences in comparison to the naked PBMD (pDNA) particle at the respective concentration is denoted as follows: *p < 0.05, **p < 0.01, and ***p < 0.001. **C** Hemolytic activity was measured as release of hemoglobin from human erythrocytes after incubation with the particles. Human erythrocytes were incubated with the particles at pH 7.4 and 6 to mimic conditions present in the blood and the endosomes respectively (n = 3 ± SD). Statistical significant differences in comparison to the naked PBMD(pDNA) particle at the respective concentration and pH value is denoted as follows: *p < 0.05, **p < 0.01, and ***p < 0.001

To evaluate the effect of the shielding polymer and the resulting changes in surface charge and stability on biocompatibility, cytotoxicity and hemocompatibility investigations were conducted (Fig. 4). Firstly, cytotoxicity was investigated in adherent (HEK293T) and suspension (K-562) cells by evaluating two different mechanims of cytotoxicity. The cells were incubated with PBMD(pDNA) particles with and without the PNC shielding polymer under similar conditions as used for transfection experiments (L/C 0.6, N/P 10, HEK293T cells 0.5–4 µg mL^−1^ pDNA and 4 + 20 h incubation; K-562 cells 0.5 – 6 µg mL^−1^ pDNA and 24 h incubation). PNAM and PCEAm homopolymers were used as controls. To asses the metabolic activity, a PrestoBlue™ assay was performed. In addition, membrane integrity was investigated by performing the CytotoxONE™ assay (Lactate dehydrogenase assay, LDH assay). Overall, both assays reveal similar trends, with cytotoxicity beeing greater in HEK293T cells where no substantial influence of shielding or control polymers was observable. In contrast, K-562 exhibited comparably high cell viabilities even at the highest tested concentrations. Furthermore, there was a clear influence of the anionic shielding polymers observable. Samples shielded with PNC and PCEAm showed substantially lower cytotoxicity in both assays with cell viabilities above 70% in comparison to the naked PBMD(pDNA) particle, especially at the highest concentrations tested. Cytotoxicity of naked PBMD(pDNA) particles in HEK293T cells is in accordance with our previous work, and can be explained by the incorporation of a high amount of cationic DMAEMA and hydrophobic monomers such as BMA [9, 39, 45].

**Fig. 5:**
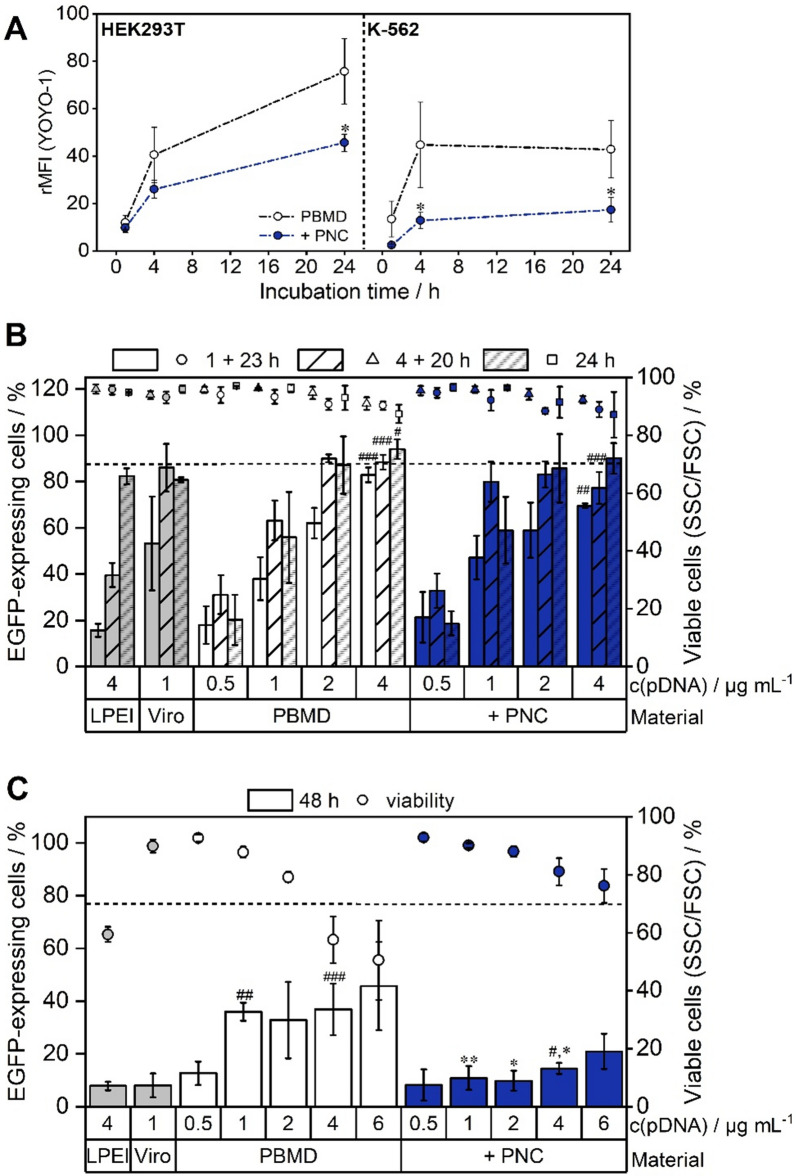
Uptake and transfection of naked and shielded particles. **A** Time-dependent uptake of PBMD(pDNA) particles with and without shielding polymer (N/P 10, L/C 0.6, 1 µg mL^−1^ YOYO-1 labeled pDNA) in HEK293T and K-562 cells. The relative mean fluorescence intensity (rMFI) of viable single cells was calculated relative to the control (particle w/o YOYO-1) (n = 3 ± SD). Statistical significant differences between naked and shielded PBMD(pDNA) particles at the respective timepoint are indicated as follows: *p < 0.05, **p < 0.01, and ***p < 0.001. **B** Transfection efficiency (percentage of viable fluorescent EGFP-expressing cells) in HEK293T cells was measured after incubation with the particles (N/P 10, L/C 0.6, 0.5–4 µg mL^−1^ pDNA) for 24 h or 1 and 4 h followed by subsequent incubation with fresh media for 24 h. **C** K-562 cells were transfected with the particles (N/P 10, L/C 0.6, 0.5–6 µg mL^−1^ pDNA) for 48 h. **B**, **C** Cytotoxicity was evaluated according to the SSC/FSC pattern (mean of n ≥ 3 ± SD). The dashed line indicates 70% cell viability. Asterisks indicate significant differences between naked and shielded PBMD(pDNA) particles at the respective timepoint and concentration: *p < 0.05, **p < 0.01, and ***p < 0.001. Significant differences in comparison to the respective control (Viromer® RED (Viro) at 1 µg mL^−1^ pDNA and LPEI at 4 µg mL^−1^ pDNA) are indicated as follows: #p < 0.05, ##p < 0.01, and ###p < 0.001

The clear impact of charge masking by shielding polymers on cytotoxicity in K-562 cells in contrast to HEK293T cells could be explained by cell type and culture conditions. Particles sedimenting over time potentially interact more strongly with adherent cells growing as a confluent monolayer. Electrostatic interactions between the nanoparticles and cells in suspension are potentially fewer, therefore not facilitating endocytosis [55]. Even when sedimenting over time, the cell surface of suspension cells interacting with the particles is less in comparison to a monolayer of adherent cells. This could result in lower uptake and cytotoxicity [44], which is also evaluated in uptake experiments below (Fig. 5, Additional file 1: Fig. S10, Table S2).

As the interaction of the shielded gene delivery system with cellular membranes is not only crucial for its biocompatibility but also for its activity as a gene delivery vector, the hemocompatibility and membrane activity at acidic pH was further investigated using human erythrocytes. Erythrocytes were incubated with naked and shielded PBMD(pDNA) particles at a L/C ratio of 0.6 and the release of hemoglobin was measured, as an indicator for membrane disruption. In order to mimic physiological and endosomal conditions, membrane activity was measured in buffer solutions at pH 7.4 and 6 (Fig. 4A). Overall, the naked PBMD(pDNA) particle exhibited a strong interaction with the erythrocyte membrane, independent of the pH value, which was not substantially influenced by the addition of the PNAM homopolymer. In contrast, the anionic shielding polymer PNC and PCEAm homopolymer significantly reduced membrane destabilization by the PBMD(pDNA) particle at physiological pH value present in blood (0.49 ± 0.1%) but revealed moderate membrane destabilization under acidification and higher concentrations (30.5 ± 17.3% at 4 µg mL^−1^ pDNA). In particular for systemic applications, the prevention of hemolytic effects under physiological conditions (pH 7.4) is of great importance, as cationic and hydrophobic moieties are known to mediate these effects, often causing side effects and reducing circulation times [7, 8, 39, 40, 56]. The PNC polymer exhibits pH- and concentration-dependent membrane protection by preventing the hemolytic effects of PBMD(pDNA) particles at neutral pH values. In contrast, the membrane interaction of PBMD(pDNA) particles is recovered at acidic pH and high concentrations. This suggests that endosomal escape is not hindered by PNC shielding and thus is not the limiting cellular barrier for transfection with this system if there is sufficient uptake of the particles to provide the concentrations required for endosomal escape.

### Cellular uptake and transfection efficiency

To further elucidate the influence of shielding on the transfection efficiency of PBMD(pDNA) particles in HEK293T and K-562 cells, cellular uptake was investigated. Therefore, the cells were incubated with YOYO-1 labeled naked and PNC shielded PBMD(pDNA) particles and measured via flow cytometry. Both cell lines exhibited YOYO-1 fluorescence after only 1 h with substantial differences in the uptake of naked and shielded particles (Fig. 5, Additional file 1: Fig. S10, Table S2). The uptake of naked particles was clearly higher in both cell lines, in particular after longer incubation times. In general, for K-562 cells lower relative mean fluorescence intensity (rMFI) values for both naked and shielded particles were observed. HEK293T cells further revealed a time-dependent uptake with increasing rMFI values over 24 h, while in K-562 the uptake reached a plateau after 4 h. This correlates with the differences observed in the PrestoBlue™ and LDH assay, with naked and shielded particles in general showing lower cytotoxicity in K-562 cells. Reduced uptake of shielded particles suggests a greater impact of charge masking in K-562 suspension cells. In addition, transfection experiments of shielded and naked PBMD(pDNA) particles were conducted in HEK293T and K-562 cells under optimized conditions for each cell line for timing of analysis after transfection and polymer concentration. EGFP-expression and cell viability were investigated for naked and PNC shielded PBMD(pDNA) particles (N/P 10, L/C ratio 0.6) at varying pDNA concentrations and incubation times (Fig. 5B, C and Additional file 1: Fig. S13). In HEK293T cells, the naked and shielded PBMD(pDNA) particles showed high transfection efficiencies, with increasing percentage of cells expressing EGFP and increasing mean fluorescence intensity (MFI) at higher pDNA concentrations and longer incubation times (4 + 20 h). Both the percentage and the MFI of EGFP-expressing cells did not increase significantly (for both naked or shielded PBMD particles) when incubation times were prolonged for up to 24 h, although the particle uptake increased (Fig. 5A, B). At shorter incubation times both particles outperformed the commonly used linear poly(ethylenimine) (LPEI) and showed similar efficiencies to Viromer® RED (Viro), a commercial polymer-based transfection agent optimized for pDNA and mRNA transfection [57]. Higher polymer concentration and longer incubation times showed little effect on viability in flow cytometry (FSC/SSC plot). However, after prolonged incubation time (24 h) and high concentrations (2 and 4 µg mL^−1^) cytotoxicity occurred as indicated by cell detachment and debris. This is in line with the toxicity screening (Fig. 3A and B) showing a decrease in metabolic activity and membrane integrity for higher concentrations already at shorter incubation times (4 + 20 h). Thus, increasing toxicity after 24 h undermines high particle uptake, which does not improve transfection efficiency. At shorter incubation times the presence of the PNC polymer did not impair transfection efficiency in HEK293T cells, and even exhibited higher transfection efficiency at 1 µg mL^−1^ pDNA compared to the naked PBMD(pDNA) particle (88.3 ± 3.2% compared to 77.2 ± 6.9%). In difficult-to-transfect K-562 cells substantial differences between naked and shielded PBMD(pDNA) particles were observed. The naked PBMD(pDNA) particle exhibited high transfection efficiencies reaching a plateau at pDNA concentrations ≥ 1 µg mL^−1^ (45.7 ± 16.8%). At the same time, substantially decreasing cell viabilities (50.4 ± 13.7% at 6 µg mL^−1^ pDNA) were observed, which are in accordance with the PrestoBlue™ assay (Fig. 4A). PNC shielded particles resulted in reduced transfection efficiency (20.9 ± 6.7% at 6 µg mL^−1^ pDNA) but considerably higher cell viability (76.1 ± 5.9%). Remarkably, both, the naked and PNC shielded PBMD(pDNA) particles outperformed the commercial standards LPEI and Viromer® RED (Fig. 5C). Thus, the PNC shielded particles demonstrate an improved biocompatibility and therefore transfection profile in comparison to the naked PBMD particles in K-562 cells.

Overall, this demonstrates that the PNC polymer has little or no influence on the transfection of HEK293T cells. This is in line with the uptake measurements, where only slight differences in uptake levels in HEK293T cells between shielded and naked PBMD(pDNA) particles following 4 h incubation (Fig. 5A) were observed. The comparatively high transfection levels of naked and shielded PBMD(pDNA) particles in HEK293T cells further complement the hemolysis results at pH 6 (Fig. 4C), showing that endosomal escape and, therefore, transfection efficiency is not impaired by the PNC polymer. However, a clear influence on transfection efficiency in case of the chronic leukemia (CML) cell line K-562 is observed. This might be as well explained by taking the hemolysis results into account (Fig. 4C). As the interaction with erythrocytes was strongly reduced at neutral pH values and cellular uptake in K-562 suspension cells was lower in the presence of PNC polymer, this might indicate that reduced membrane interaction at neutral pH is the reason for reduced transfection efficiency in suspension cells. The naked PBMD(pDNA) particle on the other hand showed high membrane interaction with erythrocytes, potentially explaining the higher cytotoxicity but also higher transfection in K-562 cells compared to the shielded particle. In this context, the PNC polymer successfully demonstrated the reduction of membrane interaction with blood and suspension cells at neutral pH, which could be beneficial for the application of the PBMD(pDNA) particle as gene delivery vector in vivo. In an initial in vivo experiment, young adult mice were intravenously injected with PNC shielded particles (PBMD(pDNA) + PNC 4 µg pDNA per mL blood volume) or with non-encapsulated (naked) pDNA as a control. Since the naked PBMD(pDNA) complex was shown to be highly hemolytic (Fig. 4C), it was not suitable as a control. Analysis of this pilot experiments at day 3 after injection revealed no overt decrease in health status of the mice that were injected with PNC shielded particles PBMD(pDNA) + PNC or as a control with naked pDNA. The injection of encapsulated pDNA achieved slightly higher percentages of transfected bone marrow blood cells compared to naked pDNA-injected mice (Additional file 1: Fig. S15, p = 0.03). However, additional experiments are needed in future studies to conclude about the superiority of PNC shielded particles for pDNA delivery and their biocompatibility. In addition, it would be important to analyze additional organ systems for measurement of transfection efficiency and cytotoxicity. As the maximal nanoparticle concentration and thus pDNA amount delivered in vivo was limited due to aggregation occurring when increasing concentrations, the nanoparticle formulation was further optimized in this regard for future in vivo studies (Additional file 1: Fig. S16–S18). Thereby, the pH responsiveness of the system, which reacts very sensitively to the smallest changes in pH, was a particular challenge. pH measurements on low concentrated PNC shielded nanoparticles (10 µg mL^−1^ pDNA) indicated that the pH range in which the nanoparticles are likely to be stable even at high concentrations is approximately pH 6.0 to 6.5 (pH 6.3). Based on this assumption, the PBMD polymer was dissolved in a slightly more acidic acetate buffer (pH 4.5), as the polymer raises the pH when dissolved at higher concentrations. This successfully resulted in PBMD(pDNA) particles with a pDNA concentration of 400 µg mL^−1^ (z-average 146.8 nm, PDI 0.210) that were in the following shielded by addition of PNC polymer dissolved in Tris buffer pH 7.0 and 7.5 (resulting pDNA concentration 200 µg mL^−1^) (Additional file 1: Fig. S 16). Addition at pH 7.5 gave slightly aggregated particles, while the particles were nicely distributed at 7.0, however with a final pH of 5.0 – 5.5 potentially already resulting in particle dissolution and thus reduced stability. The use of PNC dissolved at pH 7.3 however resulted in nicely distributed shielded particles (z-average 145.9 nm, PDI 0.247) with a final pH of 5.5 to 6.0 (Additional file 1: Fig. S17). The pDNA concentration could be even further increased to 500 µg mL^−1^ for naked PBMD particles (z-average 174.7 nm, PDI 0.214) and 250 µg ml^−1^ for the shielded particles using PNC dissolved at pH 7.4 (146.1 nm, PDI 0.239) (Additional file 1: Fig. S18). These formulation experiments demonstrate the high pH-sensibility and thus need for careful formulation optimization in non-covalent charge-based pH-responsive shielding systems when increasing particle concentration. The optimized high concentrated nanoparticle formulation now provides the possibility to apply higher pDNA amounts in future in vivo experiments to explore the full potential of the PNC shielded PBMD particles as gene delivery vector.

## Conclusions

Within this study, a non-covalent pH-sensitive shielding approach for charge masking of highly membrane active cationic hydrophobic polymer particles for gene delivery was developed. The multicomponent gene delivery system is based on a cationic hydrophobic particle core formed by the PBMD polymer, which is insoluble under physiological conditions (pH 7.4) and thus forming highly stable pDNA loaded nanoparticles, which only dissolve under acidic conditions. The core particle is non-covalently shielded by a pH-responsive block copolymer composed of an anionic pH-responsive block interacting electrostatically with the cationic core particle and a “stealthy” PNAM block, which was shown to represent an alternative to PEG. Physicochemical characterization of shielded pDNA-loaded particles revealed a well-defined particle population in the size range favored for controlled cellular uptake and nanomedical applications. The shielding polymer further showed strong interaction with the cationic particle core at blood pH (pH 7.4) preventing unspecific membrane interaction and hemolysis, without impairing pDNA complexation. When acidified these interactions are reduced, exposing the membrane-active core polymer, therefore facilitating membrane interactions beneficial for endosomal escape and efficient transfection. Biological investigations revealed high levels of uptake and transfection efficiency in adherent HEK293T cells, which was not impaired by the shielding polymer. In K-562 suspension cells, a slightly reduced percentage of transfected cells was observed, which, at the same time, showed high viabilities. Shielded PBMD(pDNA) particles thus resulted in an overall improvement of biocompatibility in K-562 cells while at the same time showing a reasonable number of transfected cells. This can be interpreted as an improved transfection profile in comparison to naked PBMD(pDNA) particles. A first in vivo testing slightly improved transfection rates in bone marrow blood cells after intravenous administration of polymer-encapsulated pDNA compared to naked pDNA while showing no overt decrease in health status of the mice. We therefore demonstrate that the biocompatibility and transfection profile of membrane active cationic hydrophobic particles can be significantly enhanced by non-covalent surface shielding with a pH-responsive stealth polymer based on PNAM, making the particles applicable in particular for the transfection of challenging suspension cells in culture and in vivo*.* Further, it was shown that the formulation can be optimized with regards to high concentrations thus offering the possibility to increase the amount of pDNA delivered in vivo to explore the particle systems full potential for in vivo gene delivery. This approach could be further optimized by the introduction of targeting units and might be furthermore applicable for immune cell-based therapies, treatment of leukemia, vaccination or gene delivery to tumor and inflamed tissue due to its pH-responsive nature.

## Supplementary Information

**Additional file 1: Table S1. **NMR integral data used to calculate conversion. **Table S2. **MFI values of different controls in flow cytometry. **Figure S1. **Characterization of CEAm monomer by ^1^H NMR. **Figure S2. **Synthesis and characterization of P(NAM_72_-*b*-CEAm_74_) (PNC) *via* RAFT polymerization. **Figure S3. **Characterization of PNAM and PCEAm homopolymers used as controls. **Figure S4.** Synthesis and characterization of PNC_DY-635_. **Figure S5.** Titration of PCEAm and PNC. **Figure S6. **CLSM study of microparticles shielded with PND_DY635_ Polymer. **Figure S7. **DNA release behavior of naked and shielded PBMD particles at pH 7.4 to pH 5 measured via the heparin release assay (HRA). **Figure S8. **DLS hydrodynamic diameter distributions and exponential decays from naked PBMD(pDNA) particles and with addition of PNC, PNAM, PCEAm. **Figure S9. **DLS and cryo-TEM measurements of PBMD and PBMD + PNC (L/C 0.6) at pH 7.4 and pH 5. **Figure S10. **Uptake of naked and shielded PBMD(pDNA) particles in HEK293T and K-562 cells. **Figure S11. **Gating strategy for uptake experiments exemplary shown for HEK293T cells. **Figure S12. **Cytotoxicity of naked and PNC shielded particles in HEK293T and K-562 cells determined by propidium iodide (PI) staining. **Figure S13. **Mean fluorescence intensity of HEK293T and K-562 cells after transfection with PBMD and PBMD + PNC (L/C 0.6) measured via flow cytometry. **Figure S14. **Gating strategy for transfection experiments exemplary shown for HEK293T cells. **Figure S15. **In vivo transfection with pDNA or pDNA encapsulated by PNC shielded particles. **Figure S16. **DLS hydrodynamic diameter distributions and exponential decays from high concentrated PBMD particles before and after addition of PNC dissolved in buffer with different pH values (pH 7.0 and 7.5). **Figure S17. **DLS hydrodynamic diameter distributions and exponential decays from high concentrated PBMD particles before and after addition of PNC dissolved in buffer with different pH values (pH 7.2 and 7.3). **Figure S18. **DLS hydrodynamic diameter distributions and exponential decays from high concentrated PBMD particles before and after addition of PNC dissolved in buffer with different pH values (pH 7.3 and 7.4).

## Data Availability

The datasets used and/or analyzed during the current study are available from the corresponding author on reasonable request.
