# Supplementary material for: pH-sensitive packaging of cationic particles by an anionic block copolymer shell
Source: J Nanobiotechnology. 2022 Jul 16;20:336. doi: 10.1186/s12951-022-01528-0 (PMC9287721; doi:10.1186/s12951-022-01528-0)
Supplement: Supplementary file 1 — Additional file 1: Table S1. NMR integral data used to calculate conversion. Table S2. MFI values of different controls in flow cytometry. Figure S1. Characterization of CEAm monomer by 1H NMR. Figure S2. Synthesis and characterization of P(NAM72-b-CEAm74) (PNC) via RAFT polymerization. Figure S3. Characterization of PNAM and PCEAm homopolymers used as controls. Figure S4. Synthesis and characterization of PNCDY-635. Figure S5. Titration of PCEAm and PNC. Figure S6. CLSM study of microparticles shielded with PNDDY635 Polymer. Figure S7. DNA release behavior of naked and shielded PBMD particles at pH 7.4 to pH 5 measured via the heparin release assay (HRA). Figure S8. DLS hydrodynamic diameter distributions and exponential decays from naked PBMD(pDNA) particles and with addition of PNC, PNAM, PCEAm. Figure S9. DLS and cryo-TEM measurements of PBMD and PBMD + PNC (L/C 0.6) at pH 7.4 and pH 5. Figure S10. Uptake of naked and shielded PBMD(pDNA) particles in HEK293T and K-562 cells. Figure S11. Gating strategy for uptake experiments exemplary shown for HEK293T cells. Figure S12. Cytotoxicity of naked and PNC shielded particles in HEK293T and K-562 cells determined by propidium iodide (PI) staining. Figure S13. Mean fluorescence intensity of HEK293T and K-562 cells after transfection with PBMD and PBMD + PNC (L/C 0.6) measured via flow cytometry. Figure S14. Gating strategy for transfection experiments exemplary shown for HEK293T cells. Figure S15. In vivo transfection with pDNA or pDNA encapsulated by PNC shielded particles. Figure S16. DLS hydrodynamic diameter distributions and exponential decays from high concentrated PBMD particles before and after addition of PNC dissolved in buffer with different pH values (pH 7.0 and 7.5). Figure S17. DLS hydrodynamic diameter distributions and exponential decays from high concentrated PBMD particles before and after addition of PNC dissolved in buffer with different pH values (pH 7.2 and 7.3). Figure S18. DLS hydrodynamic diamet [file 12951_2022_1528_MOESM1_ESM.docx]

Supporting Information

pH-Sensitive Packaging of Cationic Particles by an Anionic Block Copolymer Shell

Jana I. Solomun,^a^ Liam Martin,^a^ Prosper Mapfumo,^a^ Elisabeth Moek,^a^ Elias Amro,^c^ Friedrich Becker,^c^ Stefan Tuempel,^c^ Stephanie Hoeppener,^a, b^ Karl Lenhard Rudolph,^c^ Anja Traeger* ^a, b^

^a^ Laboratory of Organic and Macromolecular Chemistry (IOMC), Friedrich Schiller University Jena, Humboldtstrasse 10, 07743 Jena, Germany.

^b^ Jena Center for Soft Matter (JCSM), Friedrich Schiller University Jena, Philosophenweg 7, 07743 Jena, Germany.

^c^ Leibniz Institute for Age Research, Fritz Lipmann Institute, Beutenbergstraße 11, Jena 07745, Germany

*Correspondence to A. Traeger (anja.traeger@uni-jena.de)

List of Tables

Table S1. NMR integral data used to calculate conversion 6

Table S2. MFI values of different controls in flow cytometry 17

List of Figures

Fig. S1. Characterization of CEAm monomer by ^1^H NMR 11

Fig. S2. Synthesis and characterization of P(NAM_72_-*b*-CEAm_74_) (PNC) *via* RAFT polymerization. 12

Fig. S3. Characterization of PNAM and PCEAm homopolymers used as controls 12

Fig. S4. Synthesis and characterization of PNC_DY-635_ 13

Fig. S5. Titration of PCEAm and PNC 13

Fig. S6 CLSM study of microparticles shielded with PND_DY635_ Polymer. 14

Fig. S7 DNA release behavior of naked and shielded PBMD particles at pH 7.4 to pH 5 measured via the heparin release assay (HRA) 15

Fig. S8. DLS hydrodynamic diameter distributions and exponential decays from naked PBMD(pDNA) particles and with addition of PNC, PNAM, PCEAm 16

Fig. S9. DLS and cryo-TEM measurements of PBMD and PBMD + PNC (L/C 0.6) at pH 7.4 and pH 5. 17

Fig. S10 Uptake of naked and shielded PBMD(pDNA) particles in HEK293T and K-562 cells 18

Fig. S11. Gating strategy for uptake experiments exemplary shown for HEK293T cells. 19

Fig. S12. Cytotoxicity of naked and PNC shielded particles in HEK293T and K-562 cells determined by propidium iodide (PI) staining 20

Fig. S13. Mean fluorescence intensity of HEK293T and K-562 cells after transfection with PBMD and PBMD + PNC (L/C 0.6) measured *via* flow cytometry 21

Fig. S14. Gating strategy for transfection experiments exemplary shown for HEK293T cells. 22

Fig. S15. In vivo transfection with naked PBMD and PNC shielded particles 23

Fig. S16. DLS hydrodynamic diameter distributions and exponential decays from high concentrated PBMD particles before and after addition of PNC dissolved in buffer with different pH values (pH 7.0 and 7.5) 24

Fig. S17. DLS hydrodynamic diameter distributions and exponential decays from high concentrated PBMD particles before and after addition of PNC dissolved in buffer with different pH values (pH 7.2 and 7.3) 25

Fig. S18. DLS hydrodynamic diameter distributions and exponential decays from high concentrated PBMD particles before and after addition of PNC dissolved in buffer with different pH values (pH 7.3 and 7.4) 26

Instrumentation

- 1. **Nuclear magnetic resonance (NMR) spectroscopy.**

^1^H NMR (300 MHz) and DEPT ^13^C (75 MHz) spectra were recorded on a Bruker AC 300 MHz spectrometer at 300 K and a delay time (d1) of 1 s for ^1^H NMR and 2 s for DEPT ^13^C. Chemical shifts (δ) are reported in ppm.

**1.2 Size exclusion chromatography (SEC).**

SEC measurements were conducted on two instruments. An Agilent 1200 series instrument was used for dimethylacetamide (DMAc)-SEC measurements. The instrument was equipped with a differential refractive index (DRI), a UV/Vis (DAD) detector, a 1 × PSS GRAM 30 Å column (300 × 0.8 mm, 10 µm particle size) and a 1 × PSS GRAM 1000 Å column (300 × 0.8 mm, 10 µm particle size). The DMAc eluent contained 0.21% (w/w) LiCl as additive. Samples were filtered with a polytetrafluoroethylene (PTFE) membrane filter (0.45 µm pore size) prior to injection and run at 1 mL min^−1^ at 40 °C. System calibration was conducted using Poly(methyl methacrylate) (PMMA) narrow standards. SEC measurements in aqueous solution (Aq-SEC) were conducted on a Jasco system. The instrument was equipped with a AS-2051 Plus autosampler, a DG-2080-53 degasser, a PU-980 pump, a RI-2031 Plus RI detector, an UVD: UV-975 (λ_max_ = 600 nm), a Jasco oven and a PSS SUPREMA guard/1000/30 Å column (10 µm particle size). The eluent contained a mixture of 0.08 M Na_2_HPO_4_/0.05 % NaN_3_ (pH 9) and the samples were run at a flow rate of 1 mL min^−1^ at an oven temperature of 30 °C. The system was calibrated using PEG standards (400-800,000 g mol^−1^). Experimental *M*_n,SEC_ and *Ð* (*M*_w_/*M*_n_) values of the samples were determined using PSS WinGPC UniChrom GPC software.

Materials and Methods

**2.1 Materials**

Β-alanine (99%), acryloyl chloride (97%), 4-Acryloylmorpholine (97%), 1,3,5-trioxane (99%) were obtained from Sigma-Aldrich (now Merck KGaA, St. Louis, MO, U.S.). 4,4′-Azobis(4-cyanovaleric acid) (ACVA) was obtained from Sigma-Aldrich (now Merck KGaA, St. Louis, MO, U.S.) and recrystallized from methanol. 2,2'-[Azobis(1-methylethyliden)]bis[4,5-dihydro-1H-imidazoldihydrochloride was obtained from FUJIFILM Wako Chemicals Europe GmbH. Organic solvents for synthesis were obtained from Sigma-Aldrich (now Merck KGaA, St. Louis, MO, U.S.) and re-distilled on-site. Chain transfer agents, 2-(Butylthiocarbonothioylthio) propanoic acid (PABTC) and 2-(Butylthiocarbonothioylthio)propanoic acid-N-hydroxysuccinimide (NHS-PABTC) were prepared following previously reported procedures.[1] DY-635 was obtained from Dyomics GmbH. The PBMD polymer was synthesized and characterized as described in our previous work.[2] All the following materials were ordered from the suppliers stated in brackets: HEK293T cells (DSMZ, Braunschweig, Germany), K-562 cells (DSMZ, Braunschweig, Germany), TC treated cell culture flasks (Greiner Bio-One International GmbH, Kremsmünster, Austria and Labsolute, Th. Geyer GmbH & Co. KG, Renningen, Germany), TC treated multiwell cell culture plates (VWR International GmbH, Darmstadt, Germany), Dulbecco’s modified eagle medium (DMEM), Roswell Park Memorial Institute 1640 medium (RPMI) and 4-(2-hydroxyethyl)-1-piperazineethanesulfonic acid) (HEPES) buffer (Biowest SAS, Nuaillé, France), fetal bovine serum (FBS, Capricorn Scientific, Ebsdorfergrund, Germany), YOYO^TM^-1 iodide (Life Technologies, Thermo Fisher, Carlsbad, CA, U.S.), Penicillin-Streptomycin and PrestoBlue™ cell viability reagent (Thermo Fisher Scientific, Waltham, MA, U.S.), CytoTox-ONE™ Homogeneous Membrane Integrity Assay (Promega, Madison, WI, U.S.), trypsin-EDTA and 0.4% trypan blue solution (Sigma-Aldrich, now Merck KGaA, St. Louis, MO, U.S.), ethidium bromide solution and Agarose-HR PLUS (Carl Roth, Karlsruhe, Germany), heparin sodium salt (Alfa Aesar, Haverhill, MA, U.S.), linear poly(ethylenimine) (LPEI, 25 kDa, Polysciences, Warrington, PA, U.S.), Viromer® RED (Lipocalyx, now BioNTech, Mainz, Germany), Neutral Lipid Orange (NLO) (Dyomics GmbH, Jena, Germany). Plasmid DNA (pDNA) encoding for enhanced green fluorescent protein (DH-pEGFP-N1; 4.7 kb; Addgene plasmid #2491; https://www.addgene.org/vector-database/2491/; RRID: Addgene_2941), Myc (pKMyc; 4.7 kb; Addgene plasmid #1940; http://n2t.net/addgene:19400; RRID: Addgene_19400) or mCherry (mCherry2-N1; 4.7 kb; Addgene plasmid #54517; http://n2t.net/addgene:54517; RRID: Addgene_54517) was isolated from *E. Coli* using a Giga plasmid kit (Qiagen, Hilden, Germany).

**2.2 Synthesis of carboxyethyl acrylamide (CEAm)**

*Β*-alanine (5.008 g, 5.62 × 10^-1^ moles) was added to a 250 mL 2-necked round-bottom flask equipped with a magnetic stirring bar. A solution of NaOH (4.55 g, 1.14 × 10^-1^ moles) in ultrapure water (100 mL) was added and the solution allowed to cool in an ice bath. Acryloyl chloride (4.65 mL, 5.72 × 10^-1^ moles) was added dropwise over 20 min, allowed to reach room temperature (RT) and left to stir for 1 h. Concentrated HCl (37 %) was added until the solution reached pH ~ 2. The aqueous layer was extracted with EtOAc (4 × 70 mL). The organic layer was dried over MgSO_4_ and the solvent removed under reduced pressure to give white crystals, which were recrystallized from EtOAc (~ 60 mL) (1.293 g, 16% yield). ^1^H NMR (300 MHz, 300 K, DMSO-d_6_, *δ*): 12.24 (br, 1 H, (−(C=O)−OH), 8.18 (t, 1 H, −(C=O)−NH−), 6.15 – 6.25 (2 × d, 9.9 Hz, 17.1 Hz, 1 H, −(C=O)−CH=CH_2_), 6.03 – 6.09 (dd, 17.1 Hz, 2.4 Hz, 1 H, −(C=O)−CH=CH_2_), 5.54 – 5.58 (dd, 1 H, 9.9 Hz, 2.4 Hz, −(C=O)−CH=CH_2_), 3.28 – 3.34 (m, 2 H, −CH_2_−NH−), 2.39 – 2.43 (t, 6.8 Hz, 2 H, −CH_2_−CH_2_−CH_2_−). ^13^C NMR (75 MHz, 300 K, DMSO-d_6_, *δ*): 173.3 (−CH_2_−(***C***=O)−OH), 165.1 (−CH−(***C***=O)−NH−), 132.1 (CH_2_=***C***H−(C=O)−), 125.5 (−***C***H_2_=C−), 35.3 (−NH−***C***H_2_−CH_2_−), 34.2 (−***C***H_2_−(C=O)−OH), 29.7). MS: [M + H]^+^ 144.06 (calculated), 144.07 (found), [M + Na]^+^ 166.05 (calculated), 166.05 (found).

**2.3 Synthesis of P(NAM_74_-*b*-CEAm_74_)-DY635 (PNC-DY635)**

P(NAM_74_-*b*-CEAm_74_)-DY635 was synthesized in a similar manner to P(NAM_72_-*b*-CEAm_74_) by introducing PABTC-NHS (47.4 mg, 1.42 × 10^-4^ moles), NAM (1506.6 mg, 1.07 × 10^-2^ moles), ultrapure water (1197.3 mg), 1,4-dioxane (266.6 mg), a 0.1 % (w/w) solution of VA-044 in ultrapure water (248.2 mg, 0.25 mg VA-044, 7.67 × 10^-7^ moles) and 1,3,5-trioxane (external NMR standard, 27.3 mg) to a 4 mL microwave vial equipped with a magnetic stirring bar. The vial was sealed, and the solution deoxygenated by bubbling argon through it for approx. 10 min. The vial was placed in an oil bath set at 70 °C and allowed to stir for 2 h. The vial was then cooled and opened, and samples were taken for NMR and SEC analysis. Monomer conversion: ≥ 98%, DMAc-SEC: *M*_n,SEC_ = 9.8 kg mol^-1^, *Ð* = 1.09. A portion of the crude PNAM_74_ (443.5 mg, 1.89 × 10^-5^ moles polymer) was transferred to a 2 mL reaction vial equipped with a magnetic stirring bar. CEAm (202.9 mg, 1.42 × 10^-3^ moles), ultrapure water (585 mg), a 0.25 % (w/w) solution of VA-044 in ultrapure water (57.0 mg, 0.14 mg VA-044, 4.41 × 10^-7^ moles) and additional 1,3,5-trioxane (8.0 mg) was added, the vial was sealed, the solution deoxygenated with argon, and placed in an oil bath set at 70 °C. Samples were taken for NMR and SEC analysis. The polymer was dialyzed against deionised water for 4 days (MWCO: 3.5 kDa), and lyophilized to give a pale-yellow solid. Aq.-SEC: *M*_n,SEC_ = 27.7 kg mol^-1^, *Ð* = 1.24. Subsequently P(NAM_74_-*b*-CEAm_74_) was labeled with DY-635. P(NAM_74_-*b*-CEAm_74_) (30.4 mg, 1.41 × 10^-6^ moles), a 2 mg mL^-1^ solution of HBTU in DMF (400 µL, 1.93 × 10^-6^ moles), a 2 mg mL^-1^ solution of DY-635-amine in DMF (390 µL, 1.06 × 10^-6^ moles), a 2 mg mL^-1^ solution of NMM in DMF (80 µL, 1.59 × 10^-6^ moles) and DMF (130 µL) were added to a vial and allowed to stir overnight in the dark. The product was dialyzed against water/MeOH (80/20) for 2 days followed by pure water (MWCO 3.5 kDa), subsequently lyophilized and analyzed by Aq-SEC (0.1 M NaNO_3_/0.05 % NaN_3_) monitoring the RI and simultaneously the UV response at 600 nm in order to prove DY-635 attachment.

**Calculations for RAFT Polymerization.**

Monomer conversion (*p*) was calculated from ^1^H NMR data by comparing the integrals of vinyl peaks (5.5-6.3 ppm) against an external reference, 1,3,5-trioxane, (~5.2 ppm (D_2_O)), before (*t*= 0) and after (*t* = final) polymerization (eq. a). Values used for conversion calculations are shown in table S1. The theoretical number-average molar mass (*M*_n,th_) was then calculated using equation b and c for PNAM and P(NAM*-b*-CEAm) respectively:

$p={DP}_{target}*\left( 1-\frac{{Int}_{t=final}}{{Int}_{t=0}} \right)$ …… (a)

$M_{n,th}\left( \frac{g}{mol} \right)=\left( Mw_{NAM}*{DP}_{target}*p \right) + Mw_{CTA}$ ……. (b)

$M_{n,th}\left( \frac{g}{mol} \right)=\left( {Mw}_{CEAm}*{DP}_{target}*p \right)+ Mw_{macroCTA}$ ……. (c)

Where *p* is the monomer conversion of each monomer, int_t=0_ and int_t=final_ are NMR integrals relative to 1,3,5-trioxane at the start and end of the polymerization respectively. DP_target_ is the target degree of polymerization of the monomer, *Mw*_NAM_, *Mw*_CEAm_, *Mw*_CTA_ and *Mw*_macroCTA_ is the molecular weight of the monomers (CEAm and NAM), CTA (PABTC) and macroCTA (PNAM), respectively.

**Table S1:** NMR integral data used to calculate conversion

|  | DP_target_ | Int_t=0_ | Int_t=final_ |
| --- | --- | --- | --- |
| PNAM | 75 | 11.7 | 0.5 |
| P(NAM*-b*-CEAm) | 75 | 7.3 | 0.3 |

**2.4 Titration**

The polymers were titrated using a Metrohm OMNIS (Metrohm, Herisau, Switzerland) integrated titration system. The polymers were dissolved in 150 mM NaCl (in ultrapure water) at a concentration of 2.5 mg mL^‑1^. 9 mL of this solution were taken, and 1 M NaOH (150-200 µL) was added to give a basic solution (pH > 11.5). The polymers were titrated against 0.1 M HCl solution to a pH value of ≈ 2. The p*K*_a_ values were estimated using the Henderson-Hasselbalch equation from equivalence points determined by the OMNIS titration software. The degree of charge was calculated directly from the titration data and plotted against the pH value.[3]

**2.5 Gel retardation assay (GRA)**

Particle stability and pDNA release subsequent to addition of the shielding polymers was investigated by agarose gel electrophoresis as described before.[2] The release of complexed pDNA was studied preparing samples at varying L/C ratios. Homopolymers of PNAM and PCEAm containing the same number of NAM/CEAm groups as the layer polymer were used as controls. Briefly, the shielded particles were prepared as described above and subsequently diluted 6:1 with green gel loading buffer (Jena Biosciences, Jena, Germany). The samples were run on a 1% (w/v) agarose gel stained with ethidium bromide (EtBr, 0.1 µg mL^-1^) at 80 V for 1 h. The gels were subsequently imaged by using a gel imager (Red™ Imaging System, Alpha Innotech, Kasendorf, Germany).

**2.6 Dynamic and electrophoretic light scattering (DLS/ELS)**

Hydrodynamic diameter and zeta potential of shielded particles at varying L/C ratios were determined using dynamic and electrophoretic light scattering (DLS/ELS, Zetasizer Nano-ZS, Malvern Instruments, Worcestershire, U.K.) as described before.[2] Briefly, the particles were prepared as described above and size measurements were conducted in 5% (w/v) glucose solution. The particles were further diluted 1:10 to 750 µL with HBG buffer (pH 7.4) for zeta potential determination at pH 7.4. Measurements at pH 7.4 and 5 were conducted by diluting the prepared polyplexes 1:2 in 50 mM acetate-HEPES buffer of the respective pH (7.4 and 5). The instrument was operated at a temperature of 25 °C with a 633 nm He-Ne laser and at a backscattering angle of 173°. The data of three measured batches are presented as mean ± SD. Exemplary decay functions and derived hydrodynamic diameter distributions are shown in the SI (Fig. S7).

**2.7** **Cryo-transmission electron microscopy (cryo-TEM)**

For cryo-TEM measurements particles were prepared in ultrapure water as described above. For measurements at pH 7.4 and 5 samples were diluted with acetate-HEPES buffer as described for DLS measurements. Imaging was performed as described before.[2] Briefly, a FEI Tecnai G² 20 transmission electron microscope was used at an acceleration voltage of 120 kV. After blotting onto a Quantifoil grid (R 2/2, Quantifoil, 9.0 µL of solutions with a concentration of 250 µg mL^-1^) utilizing a Vitrobot Mark IV preparation unit (blotting time 1 s, offset -6 mm), the samples were vitrified using liquid ethane, maintaining the sample temperature below -175 °C at all times. The Gatan cryo transfer stage was used to load the grids into the cryo-holder (Gatan 626) and a 4k CCD camera Eagle Nanoimaging Services Inc.) was used for acquiring the images. Image processing included cropping and contrast adjustments and was performed by ImageJ (Version 1.52a, National Institutes of Health, Bethesda, MD, U.S.).

**2.7 Cytotoxicity assays**

Cytotoxicity was evaluated using two different assays assessing various mechanisms of cytotoxicity. Therefore, the PrestoBlue^TM^ assay measuring metabolic activity was compared with the CytotoxOne™ assay measuring LDH release which is related to membrane integrity. The assays were performed as described previously with slight adaptions of the procedures.[2, 4] For the PrestoBlue™ assay, HEK293T cells were seeded into 24-well plates incubated for 24 h and directly treated with the samples without a media change prior to treatment. K-562 cells were seeded as described in the main text. For treatment 50 µL of particles (N/P 10 with and without PNC polymer at L/C 0.6) prepared with pkMyc pDNA were added to test a concentration range of 0.5 - 4 µg mL^‑1^ pDNA in HEK293T cells and up to 6 µg mL^-1^ in K-562 cells. HEK293T cells were incubated for 4 h with the samples and subsequently the supernatant was removed and replaced with 500 µL D10 + H. K-562 cells were incubated with the samples or 24 h. Subsequently PrestoBlue^TM^ solution was added directly to the wells obtaining a dilution of 1:10 in the well. The cells were incubated for 45 min at 37 °C and the supernatant was transferred to a 96-well plate (100 µL per well) to measure fluorescence intensity (λ_Ex_ = 560 nm, λ_Em_ = 590 nm). Cells treated with glucose were used as a negative control, and the viability was calculated relative to the negative control after subtracting the blank (PrestoBlue^TM^ diluted in medium 1:10 without cells) using equation S1 (eq. S1)

Rel. metabolic activity / % $=\frac{\mathrm{FI}_{\mathrm{Sample}}-\mathrm{FI}_{0}}{\mathrm{FI}_{neg. control}-\mathrm{FI}_{0}} \cdot100$ (eq. S1)

where FI_sample_, FI_0_ and FI_neg. control_ represent the fluorescence intensity of a given sample, the blank and the negative control, respectively.

For the CytotoxOne™ assay the cells were seeded and treated as described above and after 24 h the supernatant was transferred to a 96-well plate (3 x 100 µL). After equilibration to RT the assay was performed according to the manufacturers protocol. Therefore, 50 µL substrate solution was added per well, incubated for 10 min at RT and the reaction was subsequently stopped by the addition of the stop solution. Fluorescence intensity was measured at *λ*_Ex_ = 560 nm / *λ*_Em_ = 590 nm. Cells treated with a 0.2% (v/v) Triton X-100 solution 30 min prior to analysis were used as positive control, while cells treated with a 5% (w/v) glucose solution were used as negative control. The relative number of cells showing LDH release and therefore a loss of membrane integrity was calculated relative to the positive control after subtracting the blank (medium without cells). By subtracting this value from 100% of cells the relative number of cells with intact membrane was calculated using equation S2 (eq. S2)

Rel. amount of intact cells / % $=100-\frac{\mathrm{FI}_{\mathrm{Sample}}-\mathrm{FI}_{0}}{\mathrm{FI}_{pos. control}-\mathrm{FI}_{0}} \cdot100$ (eq. S2)

where FI_sample_, FI_0_ and FI_pos. control_ represent the fluorescence intensity of a given sample, the blank, and the positive control, respectively.

Data were fitted in OriginPro2018b with either a logistic or linear function. Data are presented as mean of n≥3 ± SD.

**2.8 Uptake of pDNA-loaded particles by HEK293T and K-562 cells**

To study the cellular uptake HEK293T and K-562 cells were treated with PBMD(pDNA) particles at N/P 10 either without or with PNC polymer at an L/C ratio of 0.6. The assay was performed as described before with slight adaptions of the procedure.[4] Prior to particle preparation pKMyc pDNA was labeled with YOYO-1 iodide (0.027 nmol per 1 µg pDNA). Subsequently the particles were prepared as described above. The cells were treated with the particles at a final concentration of 1 µg mL^-1^ pDNA per well. Therefore 50 µL of the sample solutions was added per well and incubated with the cells for 1, 4 and 24 h. Following incubation HEK293T cells were detached by trypsin-EDTA and resuspended in the preserved cell culture supernatant and K-562 cells were resuspended for flow cytometry measurements. Trypan Blue solution was added to the cell suspension at a final concentration of 0.04% to quench fluorescence of particles attached to the cell surface, which were not taken up by the cells. The YOYO-1 signal was measured *via* flow cytometry (CytoFlex S, Beckmann Coulter, Brea, CA, U.S.) at λ_Ex_ = 488 nm combined with a 525/40 nm bandpass filter (FITC channel). Viable cells were identified by gating according to the FSC/SSC pattern. Viable single cells showing increased YOYO-1 fluorescence were identified by gating to the negative control (cells treated with PBMD particle w/o YOYO-1) and are presented as % of cells showing uptake. The relative mean fluorescence intensity (rMFI) of all viable single cells was calculated relative to the negative control. A detailed gating strategy is shown Fig. S11.

**2.9 pDNA transfection of HEK293T and K-562 cells**

Transfection experiments were conducted with HEK293T cells and K-562 cells as described before with slight adaptions of the procedure.[2, 4] Briefly, PBMD(pDNA) particles were freshly prepared as described above using DH-pEGFP-N1 pDNA and pKMyc pDNA (not encoding for green fluorescent protein) as negative control. The particles were prepared at N/P 10 with and without PNC polymer at an L/C ratio of 0.6 (final pDNA concentration in well 0.5 to 4 µg mL^-1^). For treatment 50 µL sample solution or glucose solution as control was added to the cells. HEK293T cells were incubated with the samples for 1, 4 or 24 h. Subsequently, the supernatant was removed, replaced by fresh D10 + H and the cells were further incubated up to 24 h. After 24 h the culture supernatant was removed, the cells were detached by trypsin-EDTA and subsequently resuspended in the preserved culture supernatant. K-562 cells were incubated with the samples for 48 h and resuspended prior to measurements. EGFP expression in both cell lines was measured *via* flow cytometry using the FITC channel. Viable single cells expressing EGFP were identified by gating to the negative control not encoding for EGFP (pKMyc-particle). A detailed gating strategy is shown in Fig. S14. Additionally, cell viability was determined once by propidium iodide (PI) staining. Therefore, the cells were incubated with the particles as described above and harvested for flow cytometry. Prior to measurement PI was added to a final concentration of 1 µg mL^-1^ and measured *via* flow cytometry (λ_Ex_ = 488 nm, 610/20 nm bandpass filter). PI-positive cells were gated to the positive control (LPEI without pDNA, final concentration of 50 µg mL^-1^) and the percentage of viable cells was calculated as the difference of PI-positive cells and 100%.

**2.10 In vivo transfection experiments**

150 µL of either naked mCherry pDNA or mCherry pDNA-loaded PBMD(pDNA) particles layered with PNC polymer at an L/C ratio of 0.6 (final concentration per mL blood volume: 4 µg mL^-1^ pDNA, 34 µg mL^-1^ PBMD polymer and 20.4 µg mL^-1^ PNC polymer) was intravenously injected into 4-5 months old mice. The mice were weighed and assessed daily. On day 3, the mice were sacrificed using CO_2_ and bone marrow cells were isolated, erythrocytes were lysed and analyzed *via* flow cytometry (LSR Fortessa, BD). Experiments were conducted as approved by the state government of Thuringia (reg. no. FLI-19-017).

**2.11 Formulation of high concentrated layered nanoparticles**

For the formulation of high concentrated PBMD(pDNA) particles shielded with PNC, the PBMD polymer was dissolved in 0.12 M acetate buffer (pH 4.5) at a concentration of 10 mg mL^-1^, diluted with 5 % (w/v) glucose solution to obtain concentrations that result in a N/P ratio of 10 within the final particle and mixed with pKMyc pDNA at a ratio of 1:2. The samples were vortexed for 10 s and incubated for 5 min at RT prior to the addition of the PNC polymer. The PNC polymer was dissolved in Tris 0.2 M Tris buffer at various pH values (7.0, 7.2, 7.3, 7.4 and 7.5) at a concentration of 10 mg mL^-1^. For preparation of shielded particles, the PNC polymer was diluted in 5 % (w/v) glucose solution to obtain a L/C ratio of 0.6. Subsequently the PBMD(pDNA) particles were added to the PNC polymer solution by slowly pipetting up and down. The shielded particles were incubated for 15 min at RT before usage. Naked PBMD(pDNA) particles had a final pDNA concentration of 400 or 500 µg mL^-1^, while the shielded particles had a final pDNA concentration of 200 or 250 µg mL^-1^. Due to the small volumes prepared, the pH of the particle formulations was roughly measured by using pH strips. Hydrodynamic diameter of high concentrated naked and shielded particles without dilution was determined by DLS (n = 1).

**
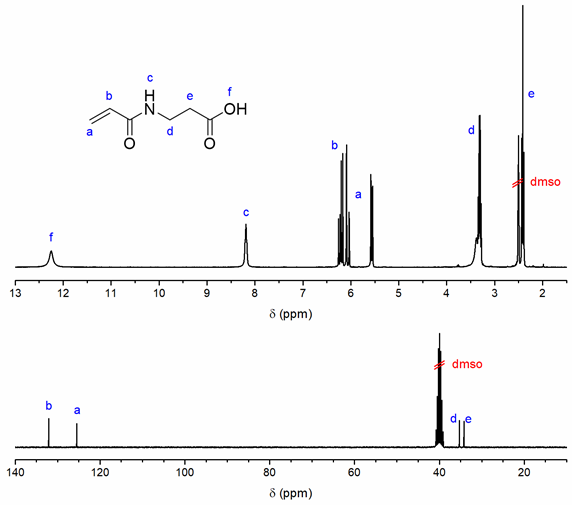
3. Further Results**

Fig. S1. Characterization of CEAm monomer by ^1^H NMR and ^13^C NMR.


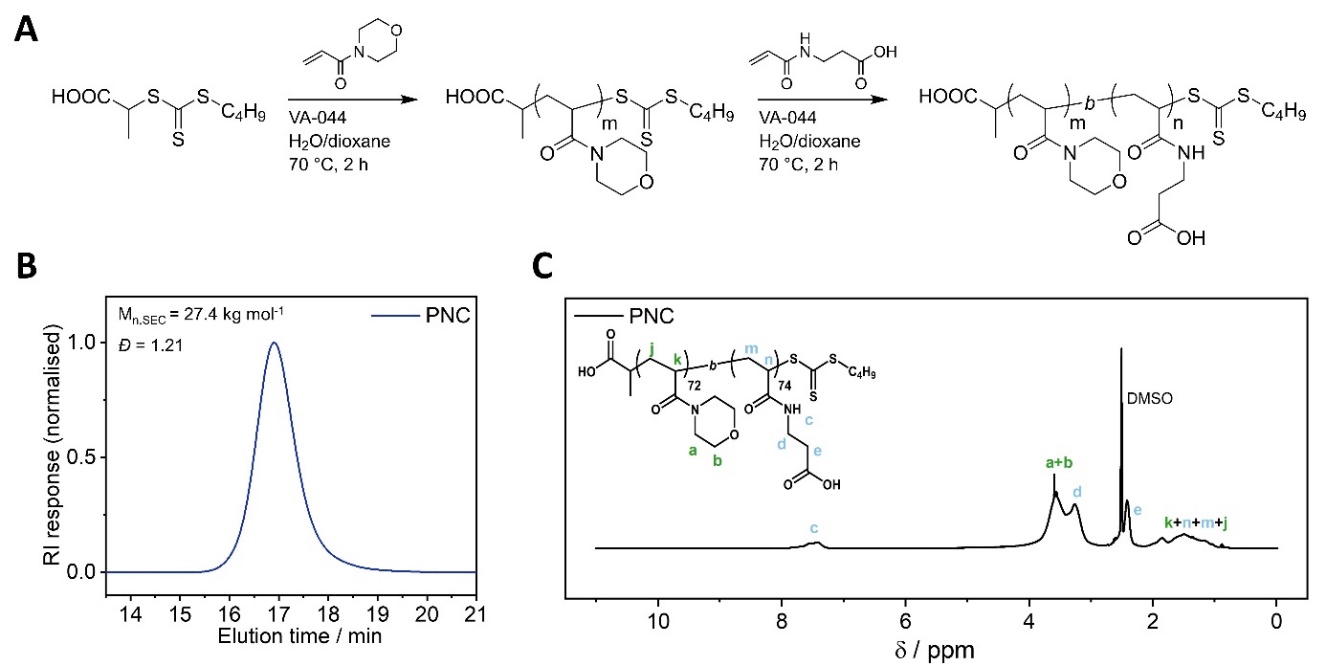
**Fig. S2.** Synthesis and characterization of P(NAM_72_-*b*-CEAm_74_) (PNC) *via* RAFT polymerization.

(A) Synthesis scheme of PNC via RAFT polymerization. (B) Characterization of the PNC block copolymer by size exclusion chromatography (SEC). PNC was characterized using aqueous SEC (Aq-SEC, 0.1 M NaNO_3_/0.05% NaN_3_) with PEG standards. (C) For further characterization ^1^H NMR was performed.


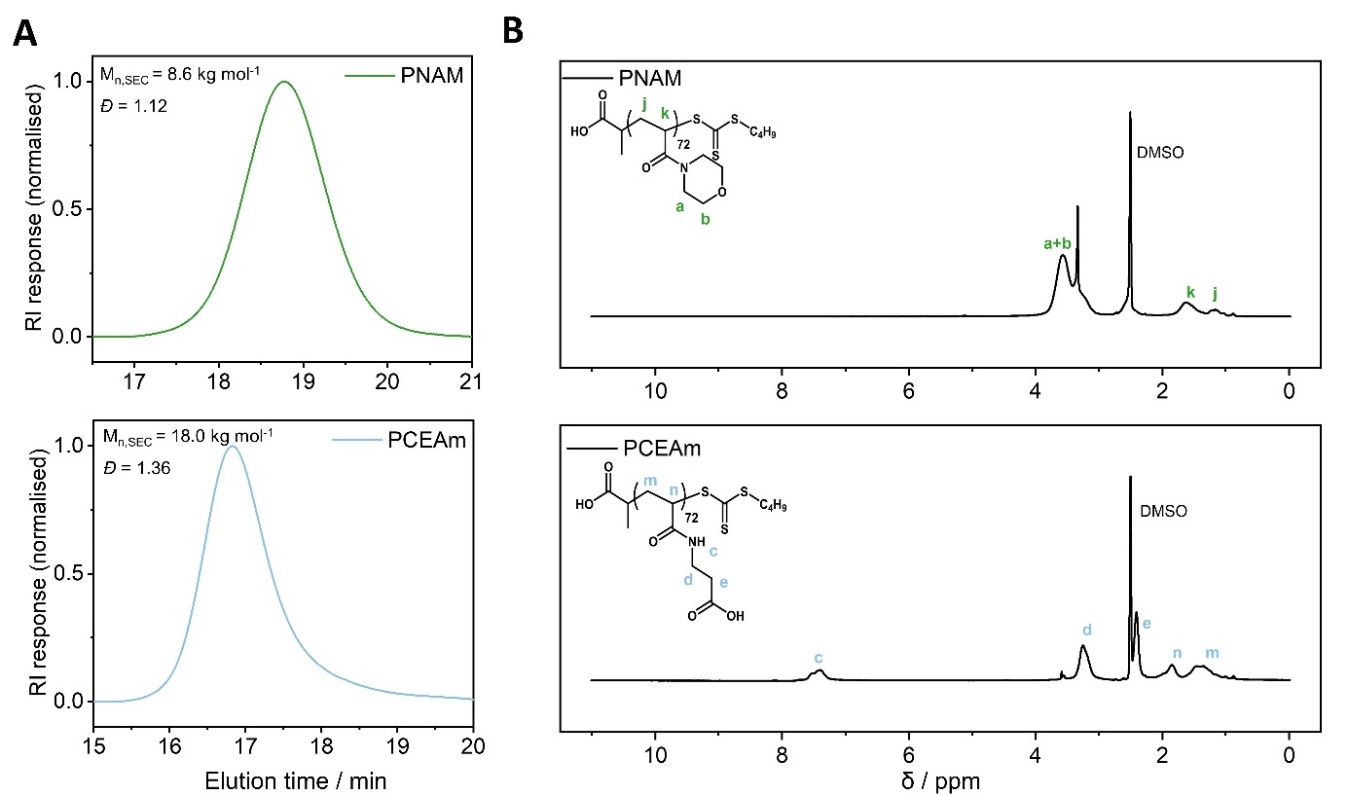


Fig. S3. Characterization of PNAM and PCEAm homopolymers used as controls.

PNAM and PCEAM were characterized by (A) size exclusion chromatography (SEC). PNAM was characterized using DMAc-SEC (DMAc + 0.21% LiCl) with PMMA standards. The anionic PCEAm homopolymer was characterized by aqueous SEC (Aq-SEC, 0.1 M NaNO_3_/0.05% NaN_3_) with PEG narrow standards. For further characterization ^1^by (B) H NMR.


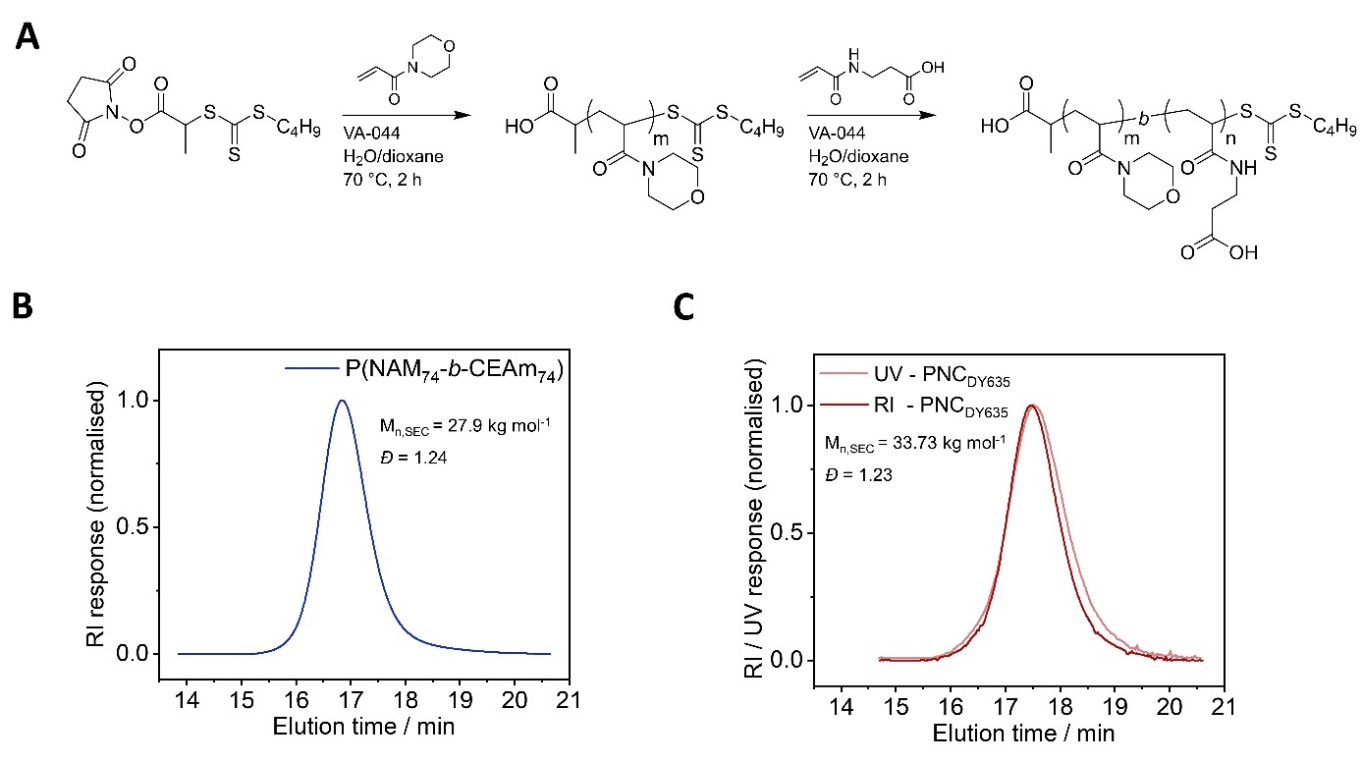


Fig. S4. Synthesis and characterization of PNC_DY-635_

(A) Synthesis scheme of P(NAM_74_-*b*-CEAm_74_) via RAFT polymerization. (B) The polymer was characterized by aqueous SEC (Aq-SEC, 0.1 M NaNO_3_/0.05 % NaN_3_) with PEG narrow standards. (C) Analysis of DY635 labeled polymer (PNC_DY635_) was performed by measuring Aq-SEC monitoring the RI and simultaneously UV response at 600 nm to prove DY635 attachment.

**
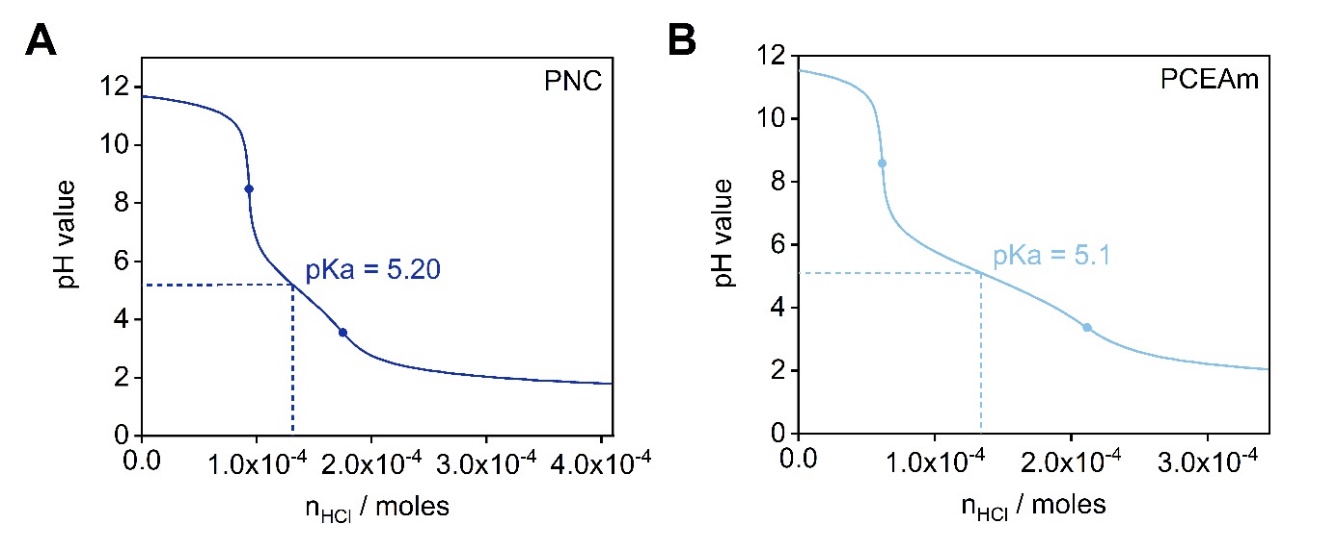
**

Fig. S5. Titration of PCEAm and PNC.

The polymers were titrated with 0.1 M HCl after dissolution in 150 mM NaCl (in ultrapure water). The p*K*_a_ values were estimated using the Henderson-Hasselbalch equation from equivalence points determined by the OMNIS titration software showing a (A) p*K*_a_ of 5.2 for the PNC polymer and (B) a p*K*_a_ of 5.1 for the PCEAm homopolymer.


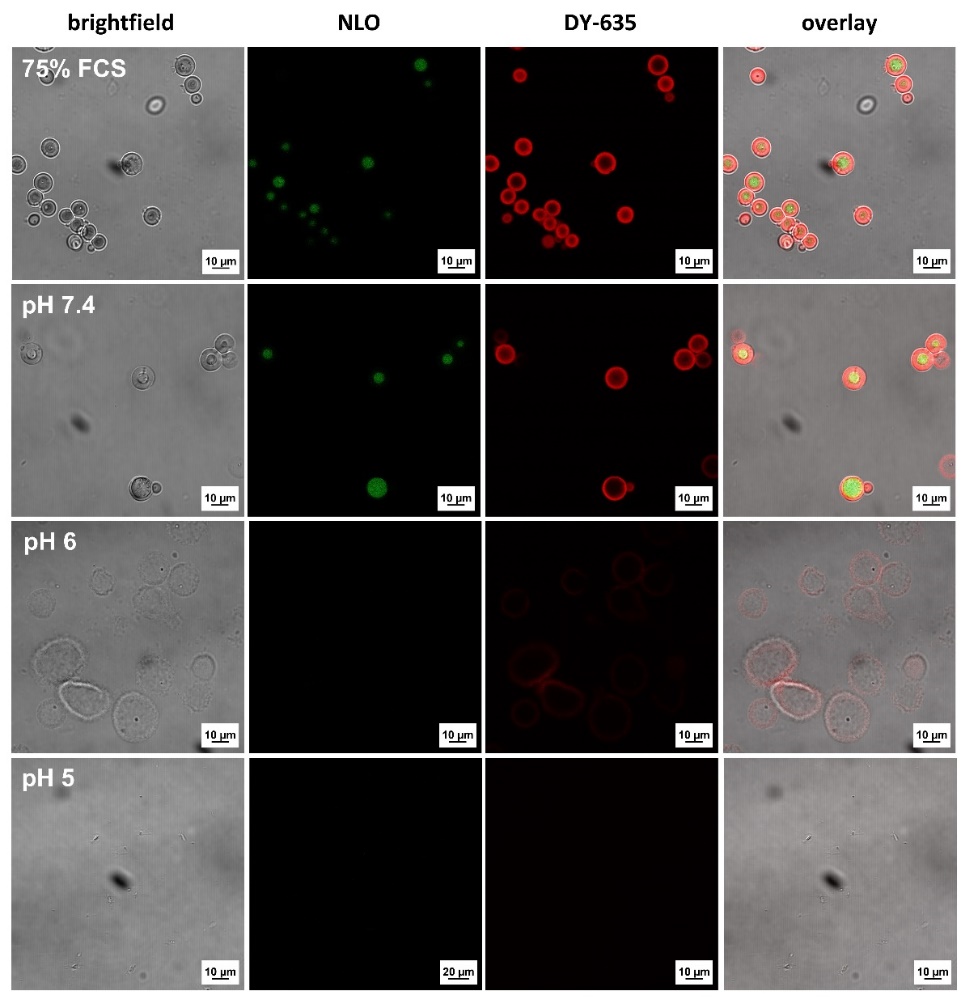


Fig. S6 CLSM study of microparticles shielded with PND_DY635_ Polymer.

PBMD-microparticles (PBMD_MP_) encapsulating neutral lipid orange (NLO, green) were prepared with PNC_DY635_ (red), incubated with buffers (HBG pH 7.4, acetate pH 6, 5) and FBS for >15 min and imaged using a confocal laser scanning microscope (CLSM). The figure shows single channels of pictures merged in the main paper.


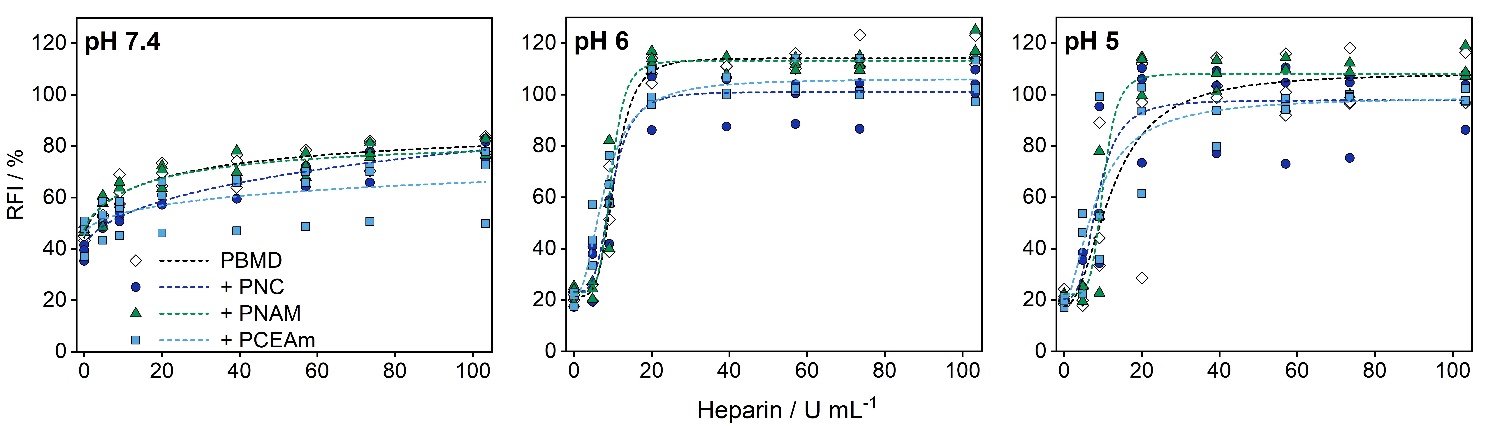


**Fig S7** DNA release behavior of naked and shielded PBMD particles at pH 7.4 to pH 5 measured *via* the heparin release assay (HRA).

The HRA was measured by addition of increasing amounts of heparin to naked and shielded particles (N/P 10; L/C 0.6) at varying pH values (HBG buffer pH 7.4, Acetate pH 6 and 5) (n = 3). The obtained data were fitted using a logistic function.

**
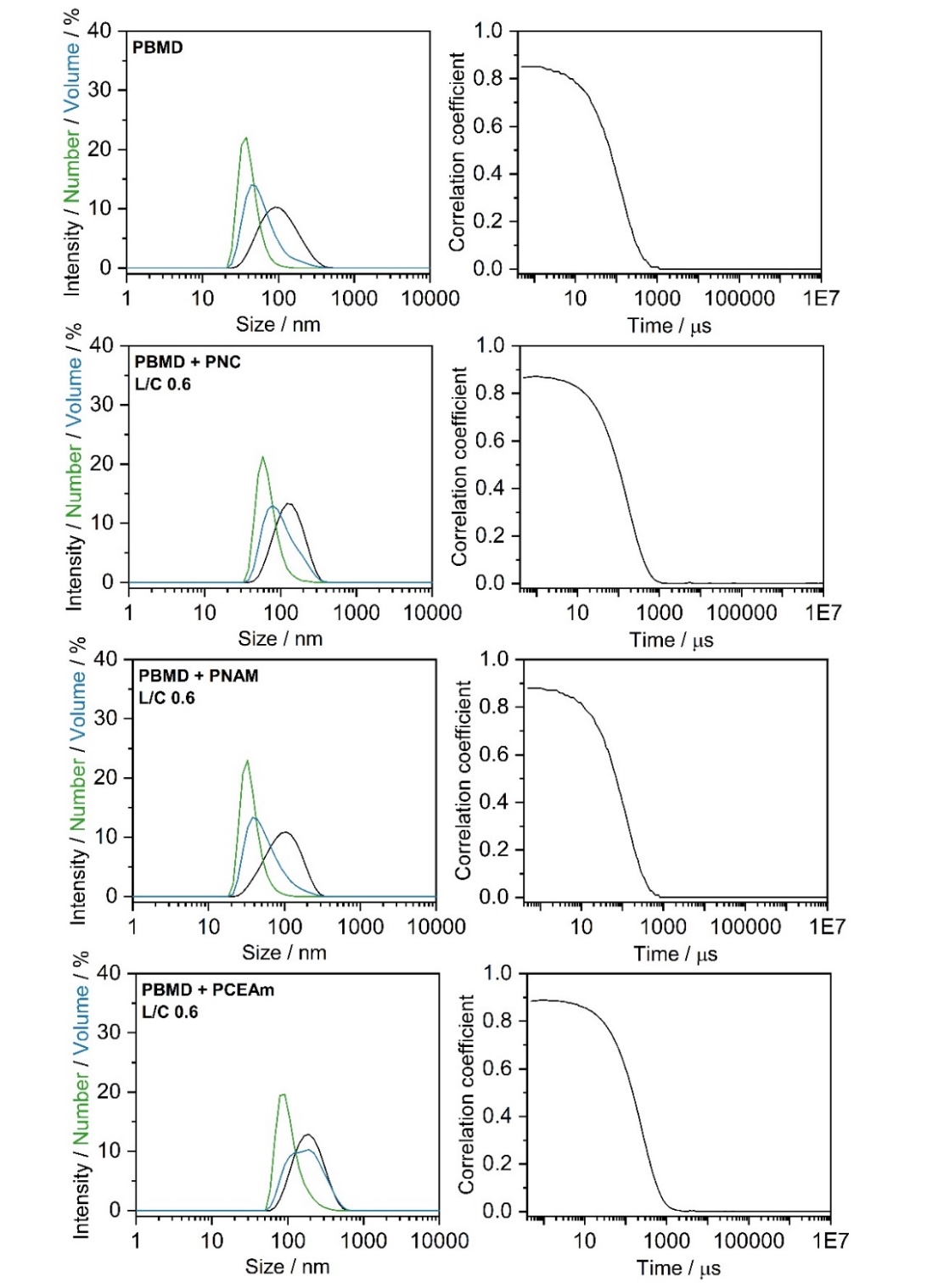
**

Fig. S8. DLS hydrodynamic diameter distributions and exponential decays from naked PBMD(pDNA) particles and after addition of PNC, PNAM, PCEAm.

Samples were measured by DLS and exemplary intensity, number, and volume weighted plots as well as exponential decay correlation coefficients of single measurements are displayed. PBMD(pDNA) particles were prepared at N/P 10 and 30 µg mL^-1^ pKMyc plasmid. PNC, PNAM and PCEAm were added to the PBMD(pDNA) particle at L/C ratios of 0.6.


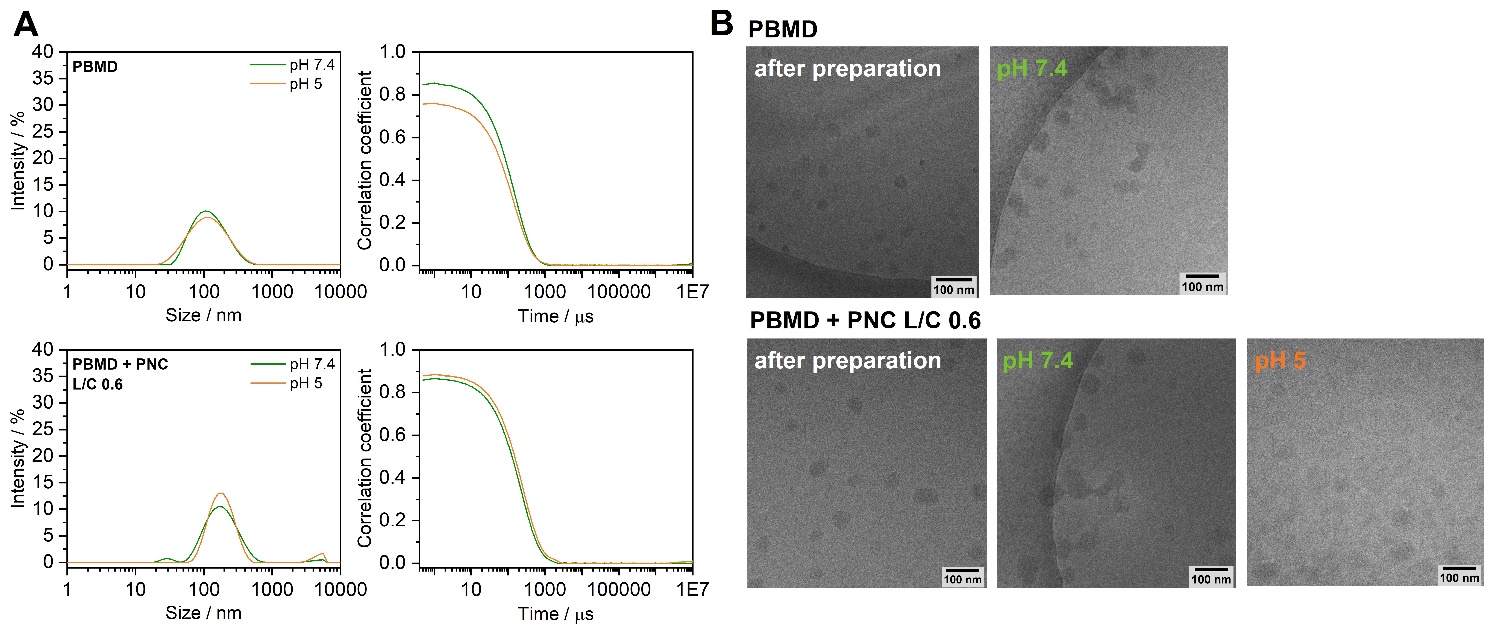


**Fig. S9** DLS and cryo-TEM measurements of PBMD and PBMD + PNC (L/C 0.6) at pH 7.4 and pH 5.

(A) Samples were measured by DLS after dilution in acetate-HEPES buffer of the respective pH (7.4 and 5) and intensity weighted plots and exponential decay correlation coefficients are displayed. (B) Cryo-TEM measurements were conducted of polyplexes after preparation and after dilution in actetate-HEPES buffer (pH 7.4 and 5).

**Table S2.** MFI values of different controls in flow cytometry.

| **Assay** | **Conditions** | **MFI of all viable single cells** | | |  |
| --- | --- | --- | --- | --- | --- |
|  |  | Only buffer | Only mEGFP pDNA/ YOYO-1 labeled pDNA | pKMyc pDNA + polymers ^[a]^ |  |
| Transfection efficiency | 4 µg mL^-1^ pDNA,  N/P 10, 1 + 23 h, **HEK293T** | 2273.04 ± 394 | 2148.04 ± 244 | 2526.74 ± 365 |  |
|  | 4 µg mL^-1^ pDNA,  N/P 10, 4 +20 h, **HEK293T** | 2402.9 ± 503 | 2227.2 ± 174 | 2990.0 ± 550 |  |
|  | 4 µg mL^-1^ pDNA,  N/P 10, 48 h, **K-562** | 1877.6 ± 143 | 1806.3 ± 94 | 2251.1 ± 116 |  |
| Uptake | 1 µg mL^-1^ pDNA,  N/P 10, 1 h, **HEK293T** | 1473.8 ± 84 | 1943.9 ± 104 | 1471.0 ± 22 |  |
|  | 1 µg mL^-1^ pDNA,  N/P 10, 4 h, **HEK293T** | 1548.2 ± 97 | 2758.2 ± 46 | 1535.3 ± 52 |  |
|  | 1 µg mL^-1^ pDNA,  N/P 10, 24 h, **HEK293T** | 1298.6 ± 19 | 2567.8 ± 209 | 1849.2 ± 94 |  |
|  | 1 µg mL^-1^ pDNA,  N/P 10, 1 h, **K-562** | 1500.0 ± 46 | 1620.2 ± 93 ± 78 | 1524.3 ± 3 |  |
|  | 1 µg mL^-1^ pDNA,  N/P 10, 4 h, **K-562** | 1521.6 ± 41 | 1929.6 ± 86 | 1577.0 ± 42 |  |
|  | 1 µg mL^-1^ pDNA,  N/P 10, 24 h, **K-562** | 1477.3 ± 120 | 2471.7 ± 374 | 1656.0 ± 98 |  |
| [a] Mean of all pKMyc pDNA-polymer polyplexes. | | | | |  |


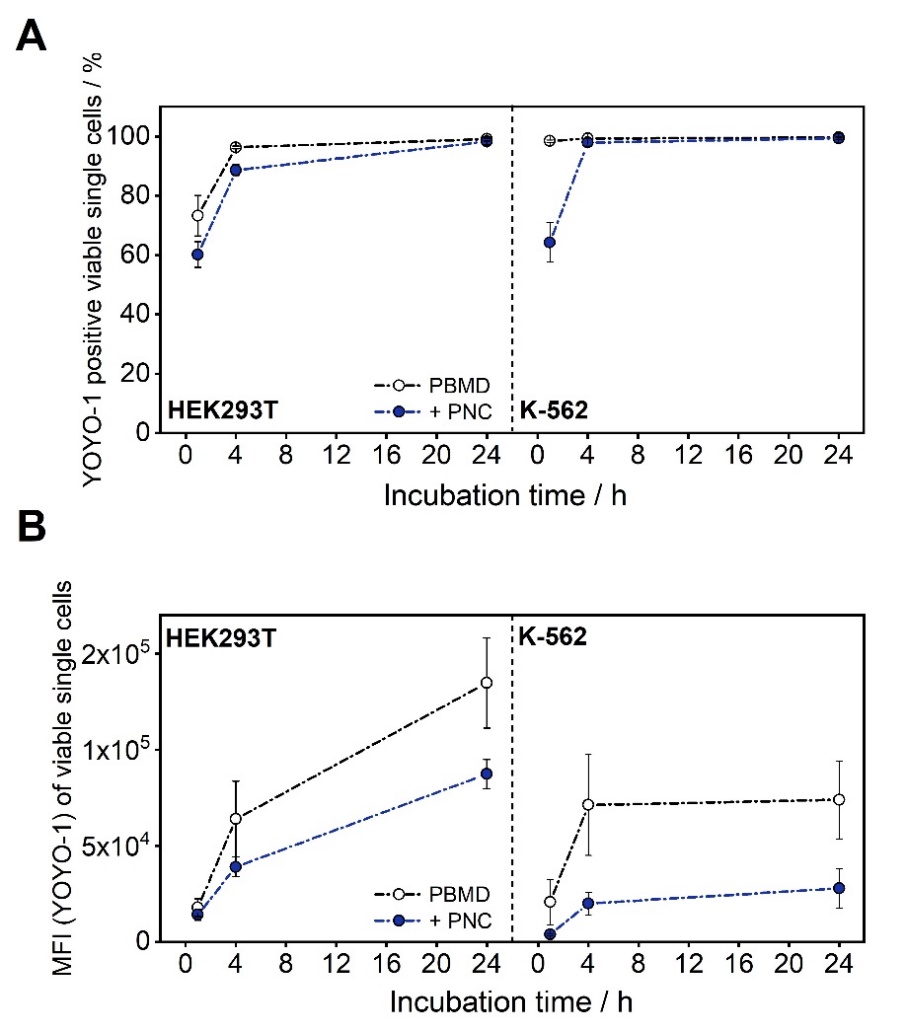


Fig. S10 Uptake of naked and shielded PBMD(pDNA) particles in HEK293T and K-562 cells.

Cellular uptake of naked and shielded PBMD(pDNA) particles (N/P 10, L/C 0.6, 1 µg mL^-1^ pDNA) was measured in HEK293T and K-562 cells after 1, 4 and 24 h *via* flow cytometry. pDNA was labeled with YOYO-1 prior to preparation of the particles. Cellular uptake is plotted as (A) percent of cells showing increased YOYO-1 fluorescence compared to the control (particle w/o YOYO-1) and (B) mean fluorescence intensity (MFI) of viable singe cells (n = 3 ± SD).

**
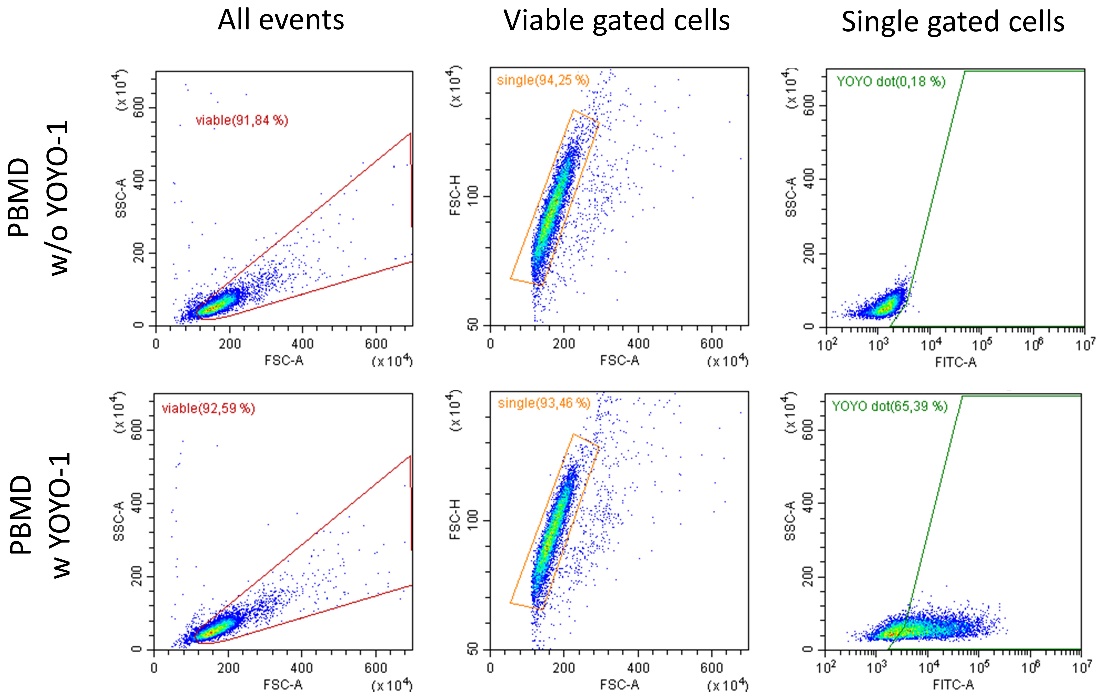
**

Fig. S11. Gating strategy for uptake experiments exemplary shown for HEK293T cells.

The gating strategy for uptake experiments is exemplary shown for HEK293T cells incubated with PBMD(pDNA) particles (N/P 10, 1 mg mL^-1^ pDNA) for 1 h. Viable cells were gated according to the FSC/SSC pattern. Viable gated cells were further gated using the area of FSC signal plotted against the FSC height (FSC-H/FSC-A plot) to discriminate single cells from doublets in the sample. YOYO-1 positive cells were identified by gating the single cells to the unstained control (cells treated with PBMD particle w/o YOYO-1).


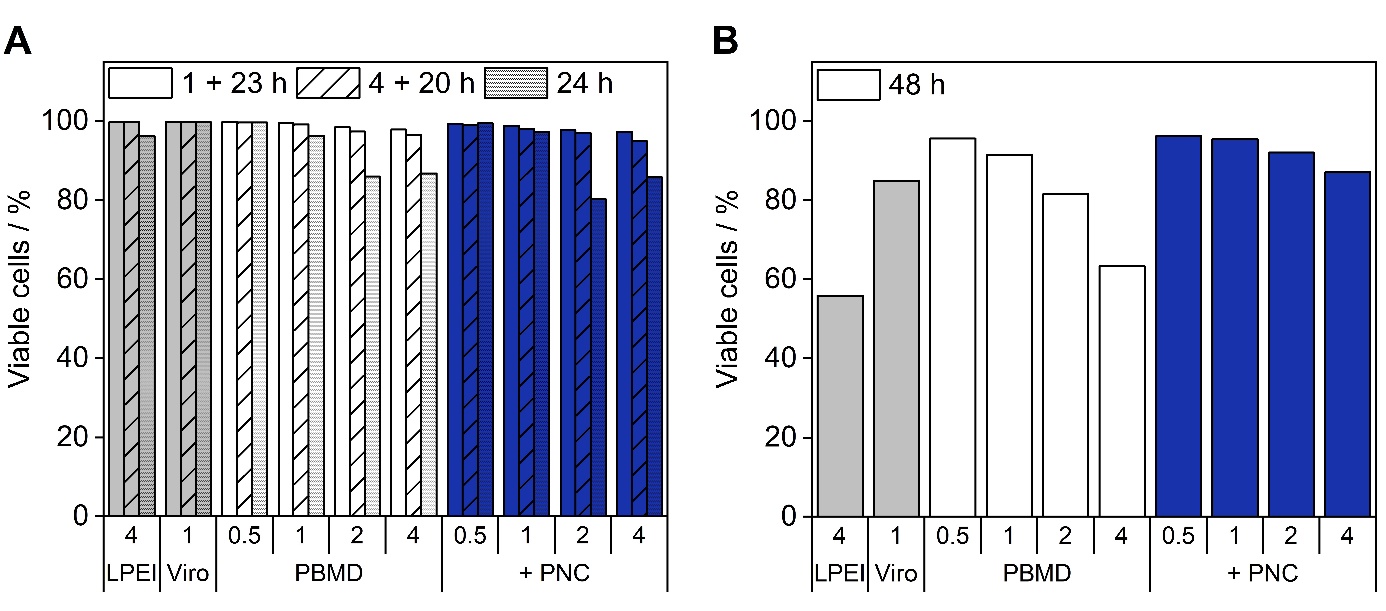


Fig. S12. Cytotoxicity of naked and PNC shielded particles in HEK293T and K-562 cells determined by propidium iodide (PI) staining.

Membrane integrity after incubation with naked and PBMD shielded particles was determined by PI staining and measured *via* flow cytometry. (A) HEK293T cells were incubated with the particles for 1 + 23, 4 + 20 or 24 h. (B) K-562 cells were incubated with the particles for 48 h. The percentage of viable cells was calculated by subtracting PI-positive gated cells from 100 % (n = 1).


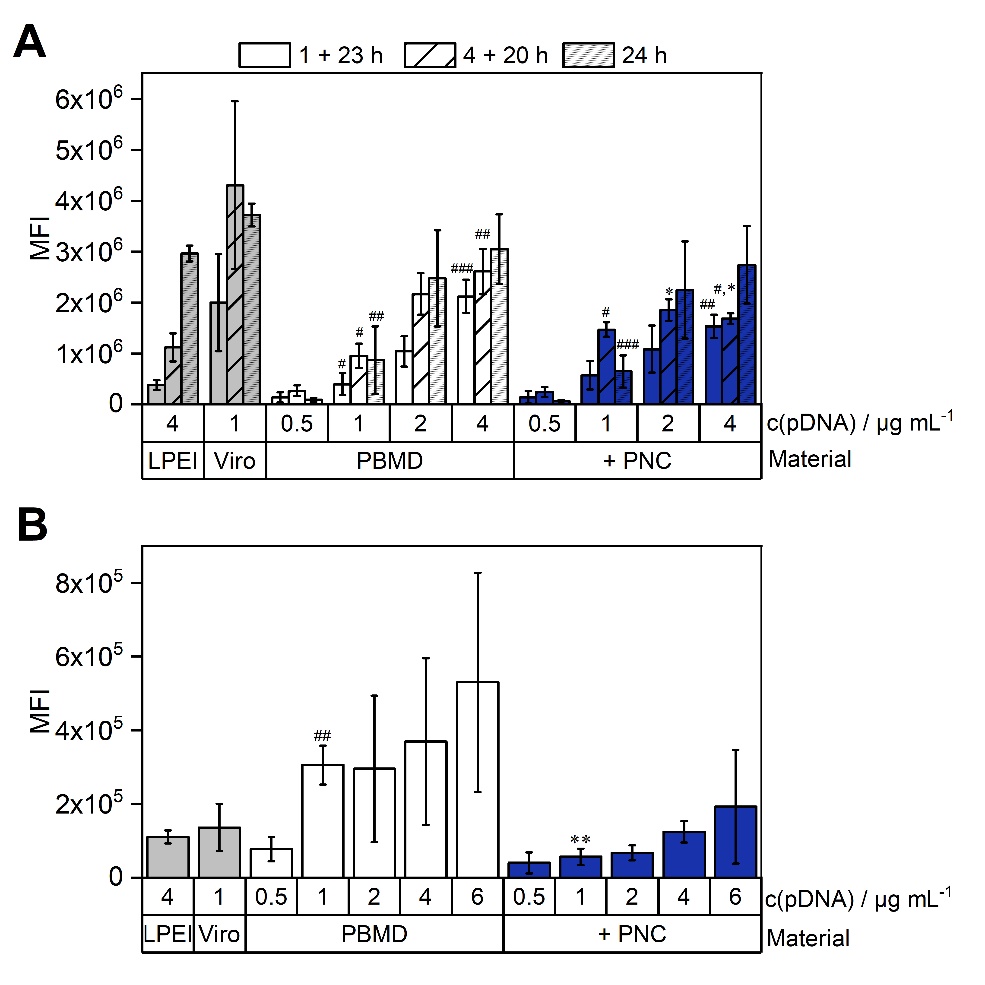


Fig. S13 Mean fluorescence intensity of HEK293T and K-562 cells after transfection with PBMD and PBMD + PNC (L/C 0.6) measured *via* flow cytometry.

(A) HEK293T cells were incubated with the particles (N/P 10, L/C 0.6, 0.5 – 4 µg mL^-1^ pDNA) for 24 h or 1 and 4 h followed by subsequent incubation with fresh media for 24 h. (B) K-562 cells were transfected with the particles (N/P 10, L/C 0.6, 0.5 – 6 µg mL^-1^ pDNA) for 48 h. Cytotoxicity (B, C) was evaluated according to the SSC/FSC pattern (mean of n ≥ 3 ± SD). The dashed line indicates 70% cell viability. Asterisks indicate significant differences between naked and shielded PBMD(pDNA) particles at the respective timepoint and concentration: *p < 0.05, **p < 0.01, and ***p < 0.001. Significant differences in comparison to the respective control (Viromer^®^ RED (Viro) at 1 µg mL^-1^ pDNA and LPEI at 4 µg mL^-1^ pDNA) are indicated as follows: # p < 0.05, ## p < 0.01, and ### p < 0.001.

**
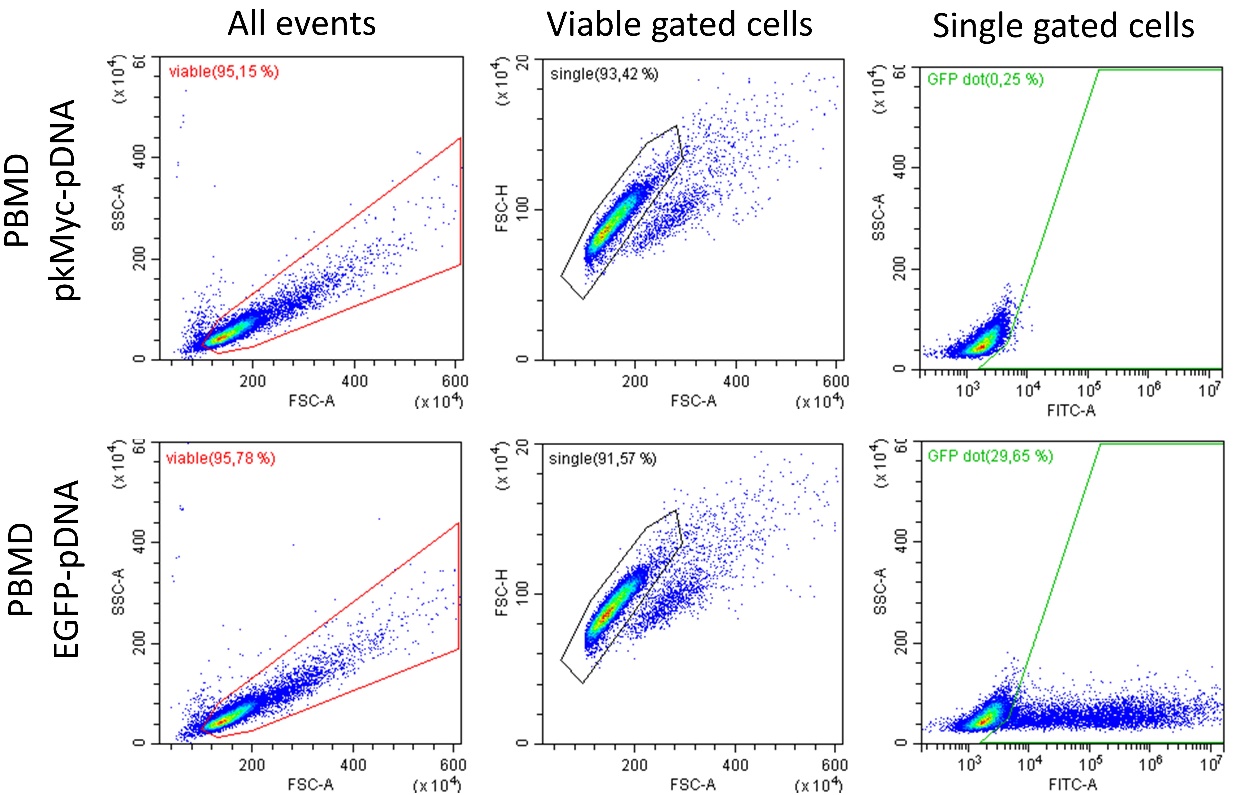
**

Fig. S14. Gating strategy for transfection experiments exemplary shown for HEK293T cells.

The gating strategy for transfection experiments is exemplary shown for HEK293T cells incubated with PBMD(pDNA) particles (N/P 10, 1 mg mL^-1^ pDNA) for 1 h and subsequent incubation in fresh media for 23 h. Viable, single cells were gated as described for uptake experiments. EGFP positive cells were identified by gating the single cells to the unstained control (polyplex with pKMyc pDNA).


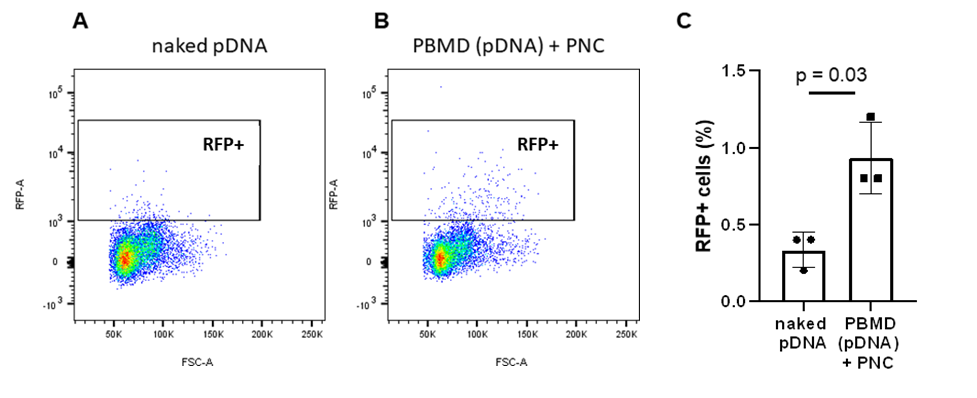


Fig. S15 In vivo transfection with naked pDNA or pDNA encapsulated by PNC shielded particles.

Four-to-five-month-old mice were intravenously injected with 4 µg (per mL blood volume) of mCherry (a red fluorescent protein = RFP) encoding DNA plasmid. The pDNA was injected without any encapsulation (naked DNA) or after polymer-encapsulation (PBMD(pDNA) + PNC). Three days after the injection, the mice were sacrificed and freshly isolated bone marrow blood cells were analyzed by fluorescence activated cell sorting (FACS) to determine the rate of targeted, RFP-positive (RFP+) cells. (A, B) Representative FACS plots of freshly isolated bone marrow cells of mice that were injected with (A) naked pDNA or (B) polymer-encapsulated pDNA (pBMD(pDNA) + PNC) are shown. The gating depicts the percentage of RFP+ cells. Gates were set based on bone marrow blood cells from non-injected control mice not showing red fluorescence-positive cells in the gate. (C) Quantification of the percentage of red fluorescence-positive bone marrow blood cells in indicated groups of injected mice is shown. Note, that the encapsulation of the pDNA increased the percentage of red fluorescence-positive bone marrow blood cells which is indicative for an increased targeting of the cells. n= 3 mice per group, unpaired Student`s t-test was used for statistical analysis.


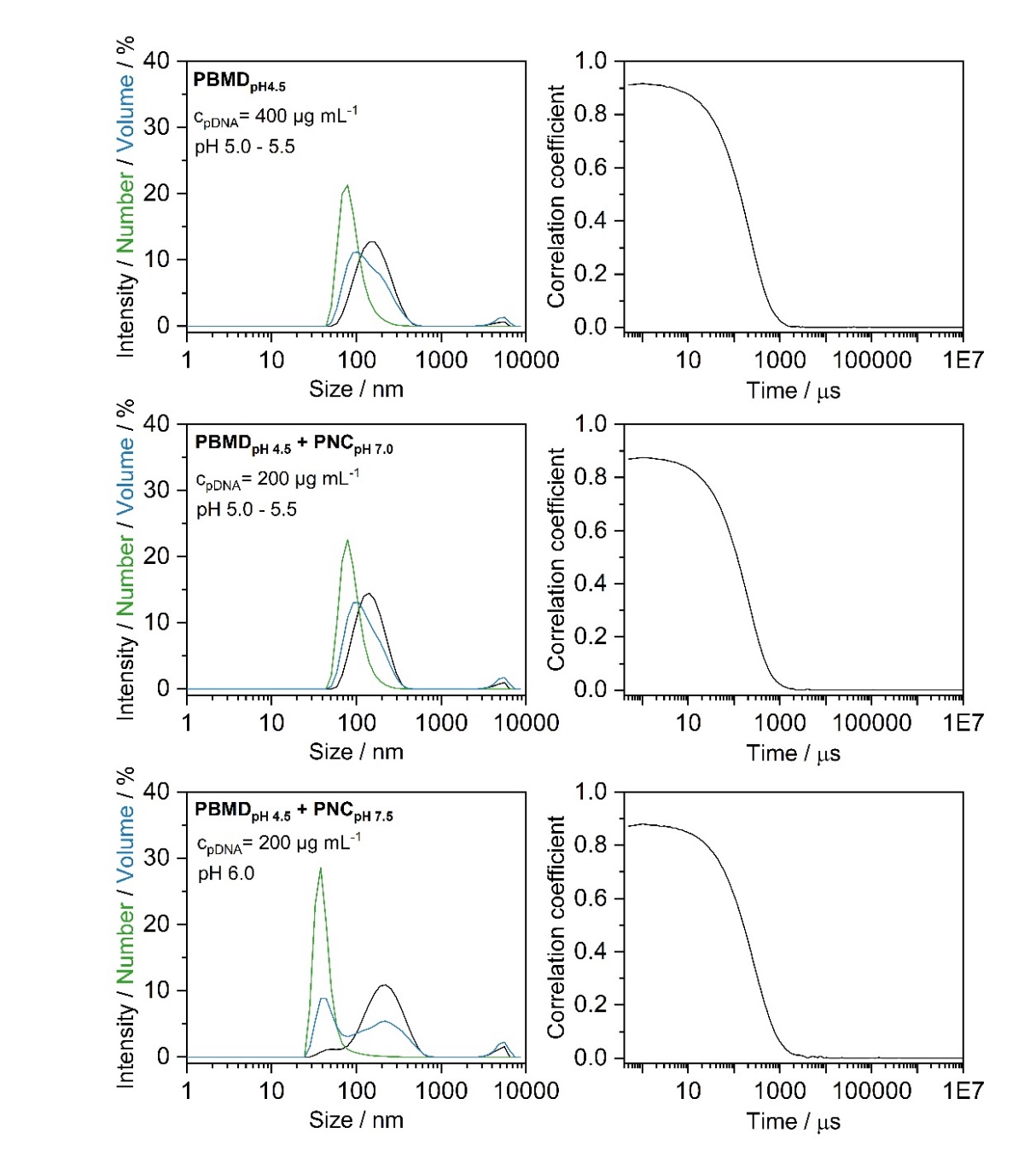


Fig. S16 DLS hydrodynamic diameter distributions and exponential decays from high concentrated PBMD particles before and after addition of PNC dissolved in buffer with different pH values (pH 7.0 and 7.5).

Samples were measured by DLS and intensity, number, and volume weighted plots as well as exponential decay correlation coefficients of single measurements are displayed. PBMD(pDNA) particles at N/P 10 were prepared with PBMD dissolved in acetate buffer (pH 4.5, 120 mM) at N/P 10 and 400 µg mL^-1^ pKMyc pDNA. PNC dissolved in Tris buffer (pH 7.0 or 7.5, 200 mM) was added to the PBMD(pDNA) particle at L/C ratios of 0.6 resulting in a pDNA concentration of 200 µg mL^-1^.


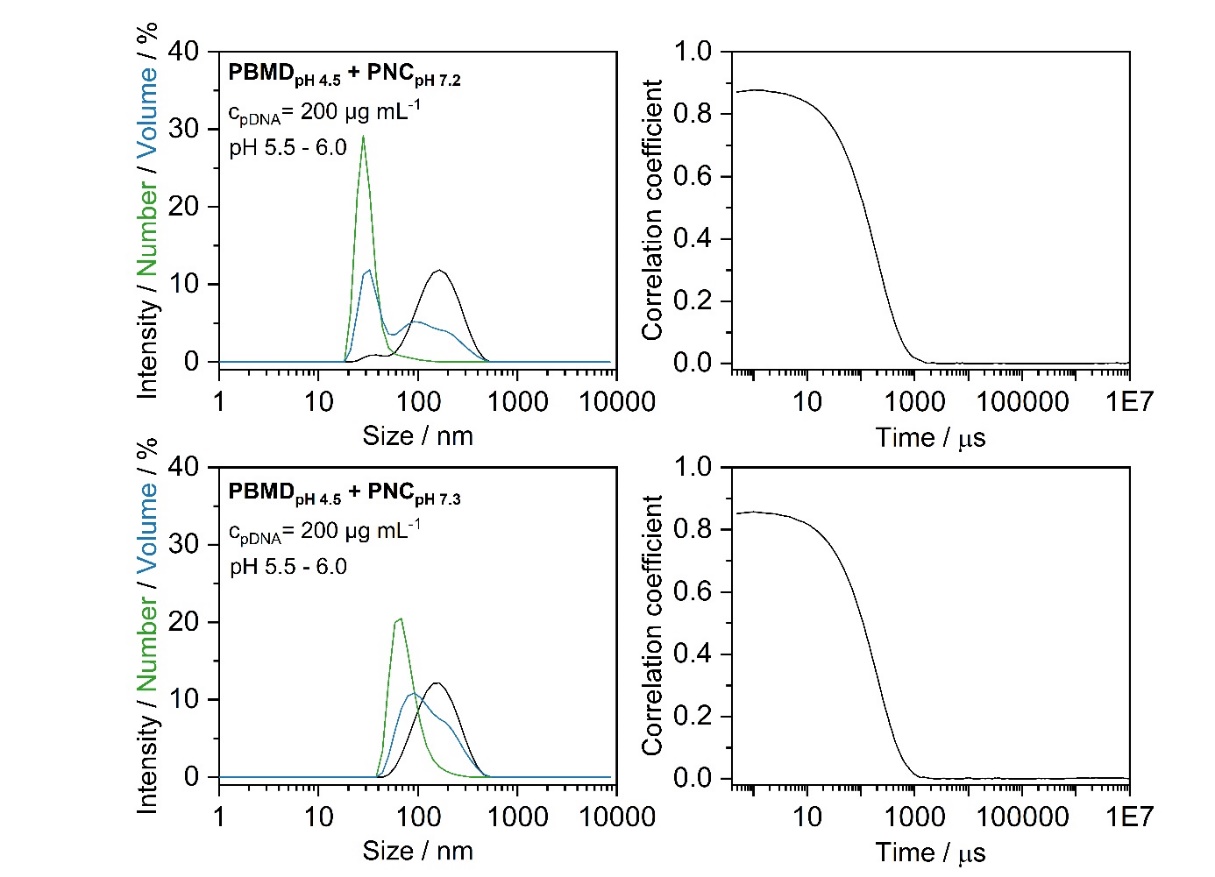


Fig. S17 DLS hydrodynamic diameter distributions and exponential decays from high concentrated PBMD particles before and after addition of PNC dissolved in buffer with different pH values (pH 7.2 and 7.3).

Samples were measured by DLS and intensity, number, and volume weighted plots as well as exponential decay correlation coefficients of single measurements are displayed. PBMD(pDNA) particles at N/P 10 were prepared with PBMD dissolved in acetate buffer (pH 4.5, 120 mM) at N/P 10 and 400 µg mL^-1^ pKMyc pDNA. PNC dissolved in Tris buffer (pH 7.2 or 7.3, 200 mM) was added to the PBMD(pDNA) particle at L/C ratios of 0.6 resulting in a pDNA concentration of 200 µg mL^-1^.


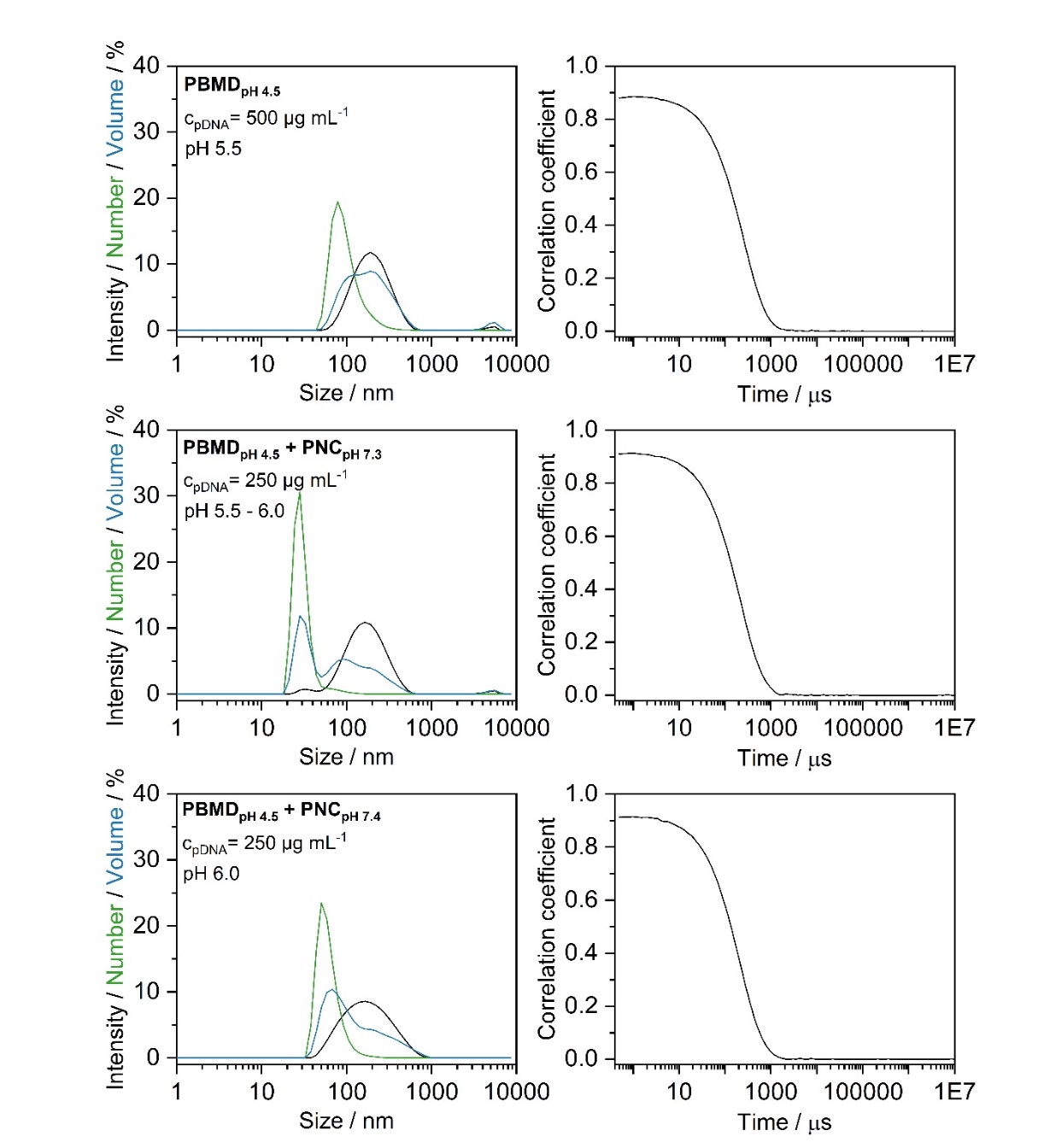


Fig. S18 DLS hydrodynamic diameter distributions and exponential decays from high concentrated PBMD particles before and after addition of PNC dissolved in buffer with different pH values (pH 7.3 and 7.4).

Samples were measured by DLS and intensity, number, and volume weighted plots as well as exponential decay correlation coefficients of single measurements are displayed. PBMD(pDNA) particles at N/P 10 were prepared with PBMD dissolved in acetate buffer (pH 4.5, 120 mM) at N/P 10 and 500 µg mL^-1^ pKMyc pDNA. PNC dissolved in Tris buffer (pH 7.3 or 7.4, 200 mM) was added to the PBMD(pDNA) particle at L/C ratios of 0.6 resulting in a pDNA concentration of 250 µg mL^-1^. The PBMD_pH4.5_ + PNC_7.4_ formulation was considered as most promising for future in vivo applications.

**4. References**

1. Larnaudie SC, Brendel JC, Jolliffe KA, Perrier S: **Cyclic peptide–polymer conjugates: Grafting‐to vs grafting‐from.** *Journal of Polymer Science Part A: Polymer Chemistry* 2016, **54:**1003-1011; doi: 10.1002/pola.27937.

2. Solomun JI, Cinar G, Mapfumo P, Richter F, Moek E, Hausig F, Martin L, Hoeppener S, Nischang I, Traeger A: **Solely aqueous formulation of hydrophobic cationic polymers for efficient gene delivery.** *International Journal of Pharmaceutics* 2021, **593:**120080; doi: 10.1016/j.ijpharm.2020.120080.

3. Colombani O, Lejeune E, Charbonneau Cl, Chassenieux C, Nicolai T: **Ionization of amphiphilic acidic block copolymers.** *The Journal of Physical Chemistry B* 2012, **116:**7560-7565; doi: 10.1021/jp3012377.

4. Richter F, Mapfumo P, Martin L, Solomun JI, Hausig F, Frietsch JJ, Ernst T, Hoeppener S, Brendel JC, Traeger A: **Improved gene delivery to K-562 leukemia cells by lipoic acid modified block copolymer micelles.** *Journal of nanobiotechnology* 2021, **19:**1-15; doi: 10.1186/s12951-021-00801-y.
